# The intra- and extracellular proteome of *Aspergillus niger *growing on defined medium with xylose or maltose as carbon substrate

**DOI:** 10.1186/1475-2859-9-23

**Published:** 2010-04-20

**Authors:** Xin Lu, Jibin Sun, Manfred Nimtz, Josef Wissing, An-Ping Zeng, Ursula Rinas

**Affiliations:** 1 Helmholtz Center for Infection Research, Inhoffenstr, 7, 38124 Braunschweig, Germany

## Abstract

**Background:**

The filamentous fungus *Aspergillus niger *is well-known as a producer of primary metabolites and extracellular proteins. For example, glucoamylase is the most efficiently secreted protein of *Aspergillus niger*, thus the homologous glucoamylase (*glaA*) promoter as well as the *glaA *signal sequence are widely used for heterologous protein production. Xylose is known to strongly repress *glaA *expression while maltose is a potent inducer of *glaA *promoter controlled genes. For a more profound understanding of *A. niger *physiology, a comprehensive analysis of the intra- and extracellular proteome of *Aspergillus niger *AB1.13 growing on defined medium with xylose or maltose as carbon substrate was carried out using 2-D gel electrophoresis/Maldi-ToF and nano-HPLC MS/MS.

**Results:**

The intracellular proteome of *A. niger *growing either on xylose or maltose in well-aerated controlled bioreactor cultures revealed striking similarities. In both cultures the most abundant intracellular protein was the TCA cycle enzyme malate-dehydrogenase. Moreover, the glycolytic enzymes fructose-bis-phosphate aldolase and glyceraldehyde-3-phosphate-dehydrogenase and the flavohemoglobin FhbA were identified as major proteins in both cultures. On the other hand, enzymes involved in the removal of reactive oxygen species, such as superoxide dismutase and peroxiredoxin, were present at elevated levels in the culture growing on maltose but only in minor amounts in the xylose culture. The composition of the extracellular proteome differed considerably depending on the carbon substrate. In the secretome of the xylose-grown culture, a variety of plant cell wall degrading enzymes were identified, mostly under the control of the xylanolytic transcriptional activator XlnR, with xylanase B and ferulic acid esterase as the most abundant ones. The secretome of the maltose-grown culture did not contain xylanolytic enzymes, instead high levels of catalases were found and glucoamylase (multiple spots) was identified as the most abundant extracellular protein. Surprisingly, the intracellular proteome of *A. niger *growing on xylose in bioreactor cultures differed more from a culture growing in shake flasks using the same medium than from the bioreactor culture growing on maltose. For example, in shake flask cultures with xylose as carbon source the most abundant intracellular proteins were not the glycolytic and the TCA cycle enzymes and the flavohemoglobin, but CipC, a protein of yet unknown function, superoxide dismutase and an NADPH dependent aldehyde reductase. Moreover, vacuolar proteases accumulated to higher and ER-resident chaperones and foldases to lower levels in shake flask compared to the bioreactor cultures.

**Conclusions:**

The utilization of xylose or maltose was strongly affecting the composition of the secretome but of minor influence on the composition of the intracellular proteome. On the other hand, differences in culture conditions (pH control versus no pH control, aeration versus no aeration and stirring versus shaking) have a profound effect on the intracellular proteome. For example, lower levels of ER-resident chaperones and foldases and higher levels of vacuolar proteases render shake flask conditions less favorable for protein production compared to controlled bioreactor cultures.

## Background

The filamentous fungus *Aspergillus niger *is well-known as a producer of primary metabolites and extracellular enzymes. For example, there are reports that glucoamylase, a starch degrading enzyme used in the food industry, is produced as extracellular protein in 20 grams per liter quantities [1]. Because glucoamylase is the most efficiently secreted protein of *Aspergillus niger*, the homologous glucoamylase (*glaA*) promoter as well as the glucoamylase signal sequence are widely used for heterologous protein production. This way the recombinant protein can be directed towards the culture medium facilitating further down-stream processing. However, limitations in the secretory pathway and proteolytic degradation often hamper the extracellular accumulation of the recombinant product. Also, regulation of *glaA *promoter controlled protein production is not fully understood, though it is clear that induction occurs when the growth medium contains starch or its degradation products maltodextrine, maltose or glucose and repression is observed when growth occurs on xylose as carbon source [2-9]. Thus, induction of *glaA *promoter controlled protein production is most often carried out by maltose addition to cultures pregrown on xylose [8,10].

Little is known about the composition of the proteome of *Aspergilli*. However, the sequenced genomes of some *Aspergilli *are now publicly available, including also the first annotated genomic sequence of *A. niger *[11] fostering now strain improvement based on integrated genomics [12]. Although annotation is far from being complete, protein identification based on peptide-mass fingerprinting is starting to become routine also for *A. niger*. Recent studies on *A. niger *proteomics are limited either to the intra- or to the extracellular proteome. A comparative study on the extracellular proteome of *A. niger *growing on different substrates has been carried out using LC/MS [13]. Moreover, the intracellular proteome of *A. niger *was analyzed under conditions leading to enhanced production of the mycotoxin fumonisin using 2-D electrophoresis and Maldi-ToF [14].

In this work, we performed a comprehensive analysis of the intra- as well as the extracellular proteome of *A. niger *growing under different culture conditions. Cultivations were carried out in well-aerated controlled bioreactor cultures as well as in shake flask culture using a defined medium with xylose or maltose as carbon source. Moreover, the time-dependent change of the extracellular proteome was also followed in response to maltose addition to a culture pregrown on xylose. Proteome analysis including the identification of individual proteins was done by 2-D gel electrophoresis combined with Maldi-ToF or Q-ToF and, additionally for extracellular proteins, with liquid chromatography followed by mass spectrometry (LC-MS) analysis based on the annotated *A. niger *genome sequence [11].

## Results and Discussion

### Catabolism of xylose and maltose by *A. niger*

**Figure 1:**
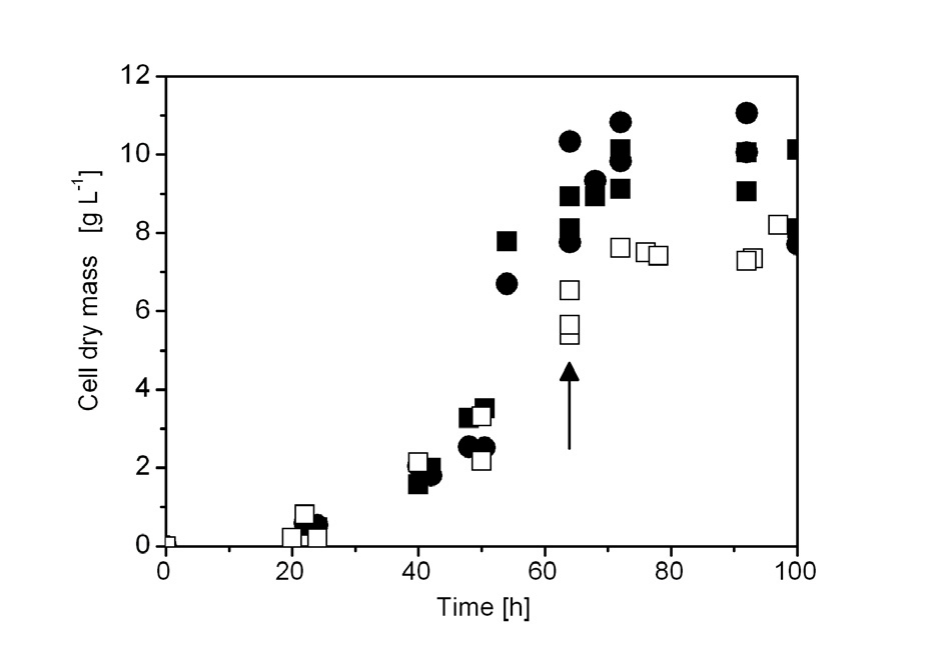
**Growth profile of *A. niger***. Growth profile of *A. niger *AB1.13 in controlled bioreactor (filled symbols) or shake flask cultures (open symbols) using either xylose (squares) or maltose (circles) as carbon substrate. Bioreactor and shake flask cultivations were carried out in duplicate and triplicate, respectively. The arrow points to the sampling point for proteome analysis.

**Figure 2:**
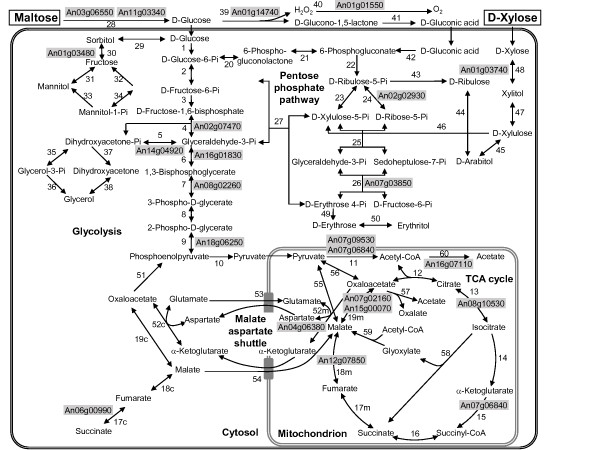
**Metabolic network of xylose and maltose catabolism**. Simplified scheme of the extra- and intracellular pathways involved in xylose and maltose catabolism in *A. niger *[64]. The reaction numbers correspond to reactions listed in Additional file 1. Proteins identified from 2-D gels (indicated by gray background) include enzymes from the glycolytic pathway: **4 **(fructose-bis-phosphate aldolase, An02g07470); **5 **(triose phosphate isomerase, An14g04920); **6 **(glyceraldehde-3-phosphate dehydrogenase, An16g01830); **7 **(phosphoglycerate kinase, An08g02260); **9 **(enolase, An18g06250); **11 **(pyruvate dehydrogenase complex: pyruvate dehydrogenase E1, An07g09530, dihydrolipoamide dehydrogenase E3, An07g06840); TCA cycle enzymes: **13 **(aconitase, An08g10530); **15 **(α-ketoglutarate dehydrogenas complex: dihydrolipoamide dehydrogenase E3, An07g06840); **18 m **(fumarase, An12g07850); **19 m **(malate dehydrogenase, An07g02160, An15g00070); enzymes from the pentose phosphate pathway: **24 **(ribose-5-phosphate isomerase, An02g02930); **26 **(transaldolase, An07g03850); enzymes from the malate-aspartate shuttle: **52 m **(aspartate aminotransferase, An04g06380); enzymes involved in anaerobic redox balancing: **17c **(cytoplasmic fumarate reductase, An06g00990); enzymes involved in polyol metabolism: **30 **(sorbitol dehydrogenase, An01g03480); acetate formation (acetyl-CoA hydrolase, An16g07110); xylose breakdown: **48 **(xylose reductase, An01g03740); and the enzymes involved in the extracellular maltose and glucose breakdown: **28 **(glucoamylase An03g06550; α-amylase, An11g03340), **39 **(glucose oxidase, An01g14740) and **40 **(catalase, An01g01550).

Xylose and maltose are both sugar carbon substrates which can be used by *A. niger *as sole carbon substrate leading to comparable growth kinetics and final biomass concentrations in controlled bioreactor cultures (Fig. 1), although both sugars are catabolized by different metabolic routes (Fig. 2). Uptake of xylose occurs in the unmodified form followed by intracellular reduction to xylitol through xylose reductase [15]. Xylitol is then converted to L-xylulose and after phosphorylation further degraded in the pentose phosphate pathway [15]. Maltose cleavage into glucose moieties occurs extracellular by glucoamylase as *A. niger *has no functional uptake system for maltose in contrast to many yeast and other filamentous fungi [16,17]. Glucose uptake occurs either directly with subsequent catabolic degradation of glucose via the glycolytic and the pentose phosphate pathway [15] or extracellular oxidation of glucose to gluconic acid by glucose oxidase with concomitant generation of hydrogen peroxide can occur as an alternative route of glucose catabolism. Gluconic acid is then further metabolized via the pentose phosphate pathway [18,19].

### Comparative intracellular proteome analysis of *A. niger *grown on xylose or maltose in bioreactor cultures

#### Intracellular proteins of equal abundance in xylose or maltose grown cultures

**Figure 3:**
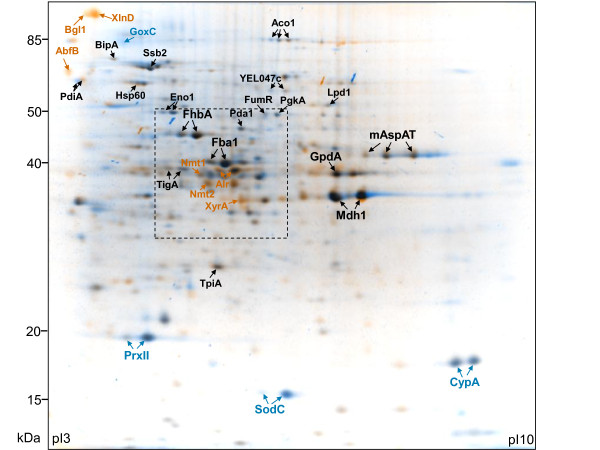
**Intracellular proteome of *A. niger *grown on xylose or maltose**. Comparative analysis of the intracellular proteome of *A. niger *AB1.13 grown to late exponential/early stationary phase in bioreactor cultures on defined medium with xylose or maltose as carbon substrate. Dual-channel image; orange and blue spots correspond to proteins which are strongly upregulated in the presence of xylose and maltose, respectively. Black spots represent proteins which are found in both cultures. Characteristics of proteins indicated by arrows are discussed in more detail. The orientation of the isoelectric focussing gel is indicated and molecular mass standards are given on the left. The framed insert indicates the section which is shown in more detail in Fig. 5. The 2-D gel images of the intracellular proteome of *A. niger *AB1.13 from xylose and maltose grown cultures with all identified proteins on interactive and searchable 2-D gels are available as Additional files 3 and 4, respectively. The complete list of proteins from the intracellular proteome of *A. niger *AB1.13 growing either on xylose or maltose, which have been identified on 2-D gels and classified into functional categories, is found in Additional file 2. A detailed list of all intracellular identified proteins showing significant changes in abundance depending on the carbon substrate is available in Additional file 5.

Despite the different routes of catabolic breakdown of the two carbon substrates, the intracellular proteome of *A. niger *growing either on xylose or maltose revealed striking similarities (Fig. 3). On both carbon substrates, the glycolytic enzymes fructose-bis-phosphate aldolase (Fba1) and glyceraldehyde-3-phosphate-dehydrogenase (GpdA), and the TCA cycle enzyme malate-dehydrogenase (Mdh1), represent the most prominent components of the intracellular proteome (Fig. 3). Furthermore, the flavohemoglobin FhbA accumulates in both cultures to high quantity. The function of this protein is not yet clear; its expression has been correlated with a hyphal growth phenotype [20] and its enzymatic characterization suggests a role in nitrosative stress protection [21]. Homologs of FhbA in *Saccharomyces cerevisiae *reveal a complex role in the oxidative stress response but no function in respiration [22,23]. This protein seems to be an important fungal protein as its homolog in *P. chrysogenum *also represents a main component of the intracellular proteome [24]. Another major protein found in both cultures in comparable abundance is the mitochondrial aspartate aminotransferase (mAspAT), a component of the malate-aspartate shuttle involved in exchanging NADH formed in the cytosol for mitochondrial NAD^+^.

Other enzymes of the central metabolic pathways, the glycolytic enzymes triose phosphate isomerase (TpiA), phosphoglycerate kinase (PgkA), and phosphopyruvate hydratase or enolase (Eno1), enzymes of the pyruvate dehydrogenase complex, namely the α subunit E1 (Pda1) and the dihydrolipoamide dehydrogenase (Lpd1, also present in the TCA cycle α-ketoglutarate dehydrogenase complex), and the other TCA cycle enzymes aconitase (Aco1) and fumarate hydratase or fumarase (FumR) and the cytoplasmic fumarate reductase (YEL047c) are found as minor spots but also of equal abundance in both cultures. Thus, enzymes of the central metabolic pathways accumulate to similar abundance in both cultures growing either on maltose or xylose as carbon source. Enzymes from central metabolic pathways identified on 2-D gels are indicated in Fig. 2. A complete list of metabolic reactions and corresponding enzymes identified on 2-D gels is found in Additional file 1.

Other important proteins present in similar amounts in the mycelium of *A. niger *grown either on xylose or maltose are ER-resident chaperones and foldases of the secretory pathway such as the DnaK-type molecular chaperones BipA and Ssb2, protein disulfide isomerases PdiA and TigA as well as heat shock proteins such as Hsp60 indicating that the maltose-induced synthesis of the extracellular glucoamylase has no influence on the amounts of ER-resident chaperones and foldases. A complete list of proteins from the intracellular proteome of *A. niger *AB1.13 grown either on xylose or maltose, which have been identified on 2-D gels and classified into functional categories, is found in the Additional file 2. Moreover, interactive and searchable 2-D gels of the intracellular proteome of *A. niger *AB1.13 from the xylose and maltose grown culture with all identified proteins indicated in Additional file 2 are found in Additional files 3 and 4, respectively.

#### Intracellular proteins found in higher abundance in the xylose-grown culture

**Figure 4:**
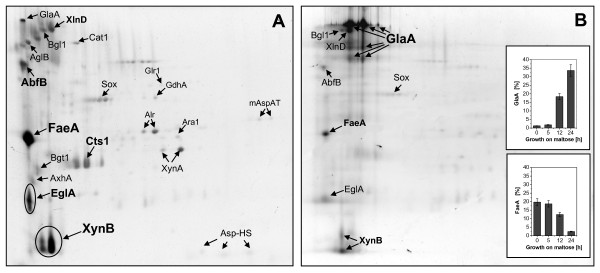
**Change of extracellular proteome in response to maltose addition to a culture pregrown on xylose**. Change of the extracellular proteome of *A. niger *AB1.13 in response to maltose addition to a bioreactor culture pregrown on xylose. **(A) **The composition of the extracellular proteome of cells grown on defined medium with xylose as carbon substrate. **(B) **Change of the extracellular proteome 24 h after the addition of maltose to a culture pregrown on xylose. Characteristics of proteins indicated by arrows are discussed in more detail. The basic side of the gel is on the right. The inserts on the right show the time-dependent fraction change of two selected proteins from the extracellular proteome after the addition of maltose. A detailed list of all proteins from the extracellular proteome of *A. niger *growing either on xylose or maltose identified on 2-D gels is found in Additional file 6.

Proteins only present during growth on xylose are D-xylose reductase (XyrA), β-xylosidase (XlnD), arabinofuranosidase B (AbfB) and β-glucosidase (Bgl1 or BglA) (Fig. 3, Additional file 5). XyrA catalyses the NADPH dependent reduction of xylose to xylitol, which represents the first step in D-xylose metabolism. Expression of the *xyrA *gene is under the control of the xylanolytic transcriptional activator XlnR [25,26]. XlnD, also under control of XlnR, catalyses the hydrolysis of xylo-oligosaccharides [26,27]. It is also present in the medium (Fig. 4), and, thus probably as cell wall associated protein present in the mycelium. AbfB and Bgl1, both not controlled by XlnR [28], are enzymes involved in the hydrolytic release of L-arabinofuranosyl residues from arabinoxylan and arabinan and in glucose moieties from gluco-oligosaccharides, respectively. Both enzymes are also found in the medium, and, thus, are probably as XlnD present as cell wall associated proteins.

Some other proteins are not exclusive to the xylose grown culture but accumulate to significantly higher amounts compared to the maltose grown culture. Noticeable examples are an NADPH dependent aldehyde reductase (Alr), and enzymes involved in the biosynthesis of thiamine, namely Nmt1 and Nmt2. The physiological function of the NADPH dependent aldehyde reductase (Alr) is yet unknown, as member of the aldo-keto reductase superfamily it may perform complex roles in carbohydrate and detoxification pathways [29,30]. Nmt1 is involved in the synthesis of thiamine, being responsible for the synthesis of the pyrimidine moiety of thiamine (vitamin B1) [31]. Nmt2 plays a central role in the biosynthesis of thiazole, a precursor of thiamine [32]. In fission yeast, it has been shown that both genes are co-ordinately controlled, highly transcribed in minimal medium and completely repressed in the presence of thiamine [32]. There are also indications that Nmt2 (Thi4 in *S. cerevisiae*) may have some role in providing oxidative damage tolerance [33,34]. A detailed list of all identified intracellular proteins showing significant changes in abundance depending on the carbon substrate is found in Additional file 5.

#### Intracellular proteins found in higher abundance in the maltose-grown culture

The only identified protein found exclusively in the maltose grown culture was glucose oxidase (GoxC) (Fig. 3, Additional file 5). This enzyme catalyzes the oxidative conversion of glucose, the product of maltose hydrolysis, into gluconic acid and hydrogen peroxide. In this line, enzymes involved in the removal of reactive oxygen species, such as superoxide dismutase (SodC) or peroxiredoxin (PrxII) were found in much higher abundance in the culture growing on maltose. An unexpected protein more abundant in the mycelium of the maltose grown culture was the protein folding catalyst cyclophilin-like peptidyl prolyl cis-trans isomerase (CypA). For a detailed list of all identified intracellular proteins showing significant changes in abundance depending on the carbon substrate see Additional file 5.

### The extracellular proteome of *A. niger *grown on xylose or maltose in bioreactor cultures

The composition of the intracellular proteome of *A. niger *growing either on maltose or xylose in controlled bioreactor cultures revealed striking similarities. The extracellular proteome, however, showed considerable differences depending on the utilisation of xylose or maltose as carbon substrate (Fig. 4 and Additional files 6 and 7).

A total of 21 proteins were identified in the extracellular proteome of *A. niger *growing on xylose by 2-D gel electrophoresis in combination with Maldi-ToF analysis (Fig. 4a, Additional file 6). The majority of these extracellular proteins are hydrolytic enzymes involved in the degradation of cell wall polymers, such as xylanolytic enzymes, namely the endo-1,4-β-xylanases A and B (XynA and XynB, respectively) and β-xylosidase (XlnD), arabinoxylan degrading enzymes such as 1,4-β-D-arabinoxylan arabinofuranohydrolase (AxhA) and arabinofuranosidase B (AbfB), as well as cellulolytic enzymes such as endoglucanase (EglA) and β-glucosidase (Bgl1). Moreover, the lactose hydrolysing α-galactosidase (AglB) and also small quantities of the starch-degrading glucoamylase (GlaA) were found in the culture medium. XynB (appearing in three different spots) and ferulic acid esterase (FaeA), an enzyme which can remove aromatic compounds (e.g. ferulic acid) from plant cell wall polysaccharides (pectin and xylan), represented the two most abundant extracellular proteins accounting for more than 30% of the entire extracellular proteome. Altogether, all extracellular hydrolases represent about 60% of the extracellular proteome of *A. niger *during growth on xylose. The majority of genes encoding these extracellular hydrolases (*xynA*, *xynB*, *xlnD, axhA*, *faeA, eglA*) are controlled by the xylanolytic transcriptional activator XlnR [26,28].

Other proteins found in the medium of *A. niger *grown on xylose are 3-β-glucanosyltransferase (Bgt1) and chitinase (Cts1). Bgt1 is responsible for the elongation of 1,3-β-glucan chains during cell wall synthesis [35] and chitinases are produced by filamentous fungi which have chitin as a cell wall component and are considered to play a pivotal role during autolysis of the fungal cell wall [36-38]. Moreover, enzymes generating or removing reactive oxygen species such as sulfhydryl oxidase (Sox), which catalyses the conversion of reduced glutathione to its disulfide form with concomitant consumption of oxygen and release of hydrogen peroxide [39], or catalase (Cat1) which protects cells against oxidative damage caused by hydrogen peroxide through its catalytic decomposition [40], were identified in the extracellular proteome of *A. niger *during growth on xylose. Moreover, a putative Asp-hemolysin (Asp-HS), a protein who's homolog in *A. fumigatus *is a virulence factor and cytolytic toxin [41], was found in the extracellular proteome during growth on xylose. Some other clearly intracellular proteins such as an NADPH dependent glutamate dehydrogenase (GdhA), glutathione reductase (Glr1), D-arabinose dehydrogenase (Ara1), the NADPH dependent aldehyde reductase (Alr) and the mitochondrial aspartate aminotransferase (mAspAT) were also detected as minor components of the extracellular proteome most likely as a result of cell lysis.

The extracellular proteome of *A. niger *grown on maltose (Additional file 7) is dominated by glucoamylase, which accounts for more than 50% of the extracellular proteome and appears in multiple spots. Components of the extracellular proteome of the maltose-grown culture which were also identified in the culture grown on xylose include, for example, β-glucosidase (Bgl1), sulfhydryl oxidase (Sox) and catalase (Cat1). Cat1 was found in much higher abundance in the extracellular proteome of *A. niger *growing on maltose. Moreover, another catalase (CatR) was additionally identified in the extracellular proteome of *A. niger *growing on maltose but not in the xylose grown culture, additionally reflecting the increased demand for removal of reactive oxygen species such as hydrogen peroxide in the maltose-grown culture. Few proteases, including small amounts of PepA, were identified in the extracellular proteome, but only in the culture grown on maltose, consistent with the fact that *A. niger *AB1.13 is a mutant protease-reduced strain which has only 1-2% of the extracellular proteolytic activity of the parental strain [42,43]. Extracellular hydrolases controlled by the xylanolytic transcriptional activator XlnR (xynA, xynB, xlnD, axhA, faeA, eglA) were completely absent in the extracellular proteome of *A. niger *AB1.13 when cells were grown on maltose (Additional file 7). A detailed list of all identified proteins from the extracellular proteome of *A. niger *growing either on xylose or maltose identified on 2-D gels or identified by nano-HPLC MS/MS (in total 69 proteins) is found in Additional file 6.

To study the change in the composition of the extracellular proteome after induction of *glaA*-promoter controlled protein production maltose was added to a culture pregrown on xylose and the change in the composition analyzed by 2-D electrophoresis (Fig. 4). The proteome changed drastically after the addition of maltose, the fraction of xylanolytic enzymes declined strongly and glucoamylase (appearing in multiple spots) began to dominate reaching a fraction of more than 30% in the secretome 24 h after the addition of maltose (Fig. 4B). Catalases did not accumulate to detectable levels in the extracellular proteome in response to the addition of maltose to the culture pregrown on xylose. This is in contrast to the increased level of catalases found in the extracellular proteome of the culture which was growing on maltose from the beginning (Additional file 7). In this line high levels of glucose and gluconate were formed in the latter while late addition of maltose to the culture pregrown on xylose did not lead to detectable formation of glucose and gluconate (data not shown).

### Comparative analysis of the intracellular proteome of *A. niger *grown on xylose in controlled bioreactor or shake flask culture

Most often *A. niger *is grown in shake flask culture without the possibility to control pH and provide sufficient oxygen through controlled airation and agitation. This difference in growth conditions can translate into different productivites most often with considerable lower product concentrations obtainable in shake flask cultures. Using the same inoculum and the same medium with xylose as carbon substrate, similar general growth kinetics were observed in bioreactor and shake flask cultures (Fig. 1). The final biomass, however, was considerably lower in shake flask culture reaching only appr. 80% of the biomass obtainable in controlled bioreactor culture.

**Figure 5:**
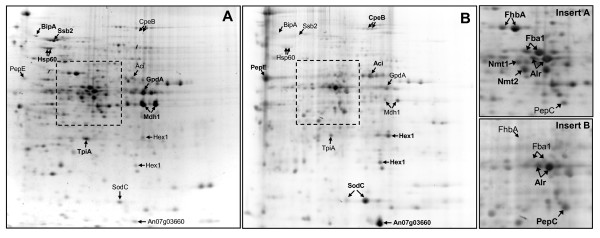
**Intracellular proteome of *A. niger *from bioreactor and shake flask grown cultures**. Comparative analysis of the intracellular proteome of *A. niger *AB1.13 grown to late exponential/early stationary phase on xylose in **(A) **batch bioreactor or **(B) **shake flask culture. Enlargements of the inserts for more details are given on the right. Characteristics of proteins indicated by arrows are discussed in more detail. The basic side of the gel is on the right. A comprehensive list of all identified intracellular proteins showing significant changes in abundance during growth on xylose in batch bioreactor or shake flask culture is available in Additional file 8.

The intracellular proteome of *A. niger *growing to late exponential/early stationary phase on the same xylose containing defined medium either in controlled batch bioreactor or shake flask culture was surprisingly different (Fig. 5, Additional file 8). Abundant proteins present in the mycelium of the bioreactor culture such as the glycolytic enzymes Fba1 and GpdA and the TCA cycle enzyme Mdh1 were only present in minor amounts in the mycelium of the shake flask culture indicating a reduced capacity of the glycolytic pathway and the TCA cycle under these conditions. Also, the flavohemoglobin FhbA and the enzymes involved in the biosynthesis of thiamine, Nmt1 and Nmt2, all prominent proteins in the bioreactor culture with putative roles in the oxidative stress response and oxidative damage tolerance, were not or only in minor amounts present in the shake flask culture probably indicating the reduced oxygen supply in shaker flasks. In shake flask culture, however, the NADPH-dependent aldehyde reductase (Alr) represented one of the major components of the intracellular proteome. Also, SodC was one of the most abundant proteins in the shake flask grown culture but only a faint spot on the 2-D gel from the bioreactor culture sample. In this line, a catalase (CpeB) was only found in the intracellular proteome of the shake flask but was not present in the intracellular proteome of the bioreactor culture indicating that cells experience more oxidative stress from reactive oxygen species (radicals and peroxides) during shake flask cultivation in contradiction to the reduced oxygen availability in shaker flask cultures. Moreover, fungal proteases such as the vacuolar proteases PepC, a subtilisin-type serine endoprotease [44], and PepE, an aspartic pepsin-like endoprotease [45], Hex1 (a structural component of the fungal Woronin body, [46]), or an NAD^+^-dependent formate dehydrogenase of yet unclear physiological function (Fdh, formerly known as AciA [47-49]) were found in higher quantities in the shake flask culture (Fig. 5, Additional file 8). The most abundant protein, however, present in the mycelium of *A. niger *growing in the shake flask culture but only present as a minor protein in the mycelium of the bioreactor culture was identified as An07g03660 (similar to An09g00630) a CipC-like protein of yet unknown function (Fig. 5, Additional file 8). The name CipC derives from *c*oncanamycin-*i*nduced *p*rotein because its orthologue in *A. nidulans *is upregulated in response to the antibiotic concanamycin A [50]. Moreover, in *A. fumigatus *CipC was identified as one of the major conodial surface associated proteins [51].

In general, glycolytic and TCA cycle enzymes and many chaperones and foldases (e.g. BipA, Ssb2, Hsp60, TpiA) accumulated to significantly higher levels in the bioreactor culture, while in shake flask cultures vacuolar proteases (PepE and PepC) and (oxidative) stress proteins involved in the removal of reactive oxygen species and other proteins of more diverse function were of higher abundance. Thus, the difference in culture conditions (pH control versus no pH control, aeration versus no aeration and stirring versus shaking) has a more profound effect on the metabolic status of the cells and the composition of the intracellular proteome than the carbon substrate. For a detailed list of all identified intracellular proteins showing significant changes in abundance during growth in bioreactor or shake flask culture see Additional file 8.

## Conclusions

### Intracellular proteome

The comparative analysis of the intracellular proteome of *A. niger *grown either on xylose or maltose in controlled batch bioreactor cultures revealed striking similarities. The glycolytic enzymes Fba1 and GpdA and the TCA cycle enzyme Mdh1 were among the most abundant proteins and accumulated to comparable amounts in both cultures. Past enzyme activity measurements in continuous cultures of *A. niger *revealed malate dehydrogenase as the most active enzyme of the TCA cycle with an activity steadily increasing with increasing dilution rate [52]. Thus, similar and elevated amounts of Mdh1 during batch growth on xylose and maltose in bioreactor culture might reflect the comparable high growth rates obtained on these substrates and indicate that energy metabolism and not the initial steps in xylose or maltose breakdown are controlling the final growth rate. In this line, a transcriptomic comparison of *A. nige*r growing either on xylose or maltose in carbon-limited continuous cultures at identical dilution rate also revealed little differences in the expression of genes from central metabolic processes [53].

Moreover, comparable high amounts of GpdA in both xylose and maltose grown batch bioreactor cultures also explain previous observations that *gpdA *promoter controlled production of recombinant proteins leads to similar amounts of the recombinant product in controlled bioreactor cultures using either glucose or xylose as carbon substrate [54].

ER-resident chaperones and foldases were present in both cultures in similar amounts indicating a comparable capacity for protein secretion under both conditions. The transcriptomic analysis of *A. nige*r growing either on xylose or maltose in carbon-limited continuous cultures indicated a modest but significant higher expression of secretory pathway genes during growth on maltose [53]. This discrepancy to our observation of equal abundance of ER-resident chaperones and foldases in both xylose and maltose batch cultures is difficult to evaluate as it might be due to the different strains and culture conditions employed. Moreover, a modest increase in transcript level also does not necessarily translate into a detectable increase in protein amount. Yet, these results clearly show the need for a combined transcriptome and proteome analysis.

Strong differences in the intracellular proteome of *A. niger *growing on xylose or maltose are related to the first steps in carbon substrate catabolism. The culture growing on xylose exclusively contained D-xylose reductase and some plant cell-wall (e.g. xylan, arabinoxylan) degrading enzymes while glucose oxidase was only found in the culture using maltose as carbon substrate. Most strikingly were the differences in the amount of intracellular fungal proteins involved in the oxidative stress response; growth on maltose was clearly leading to higher levels of enzymes involved in the removal of reactive oxygen species indicating higher oxidative stress during maltose/glucose breakdown.

### Extracellular proteome

The composition of the extracellular proteome was completely different for both bioreactor cultures. *A. niger *growing on xylose secreted mainly hydrolases involved in the degradation of plant cell wall polymers while the extracellular proteome of *A. niger *growing on maltose was dominated by glucoamylase. Again, enzymes involved in the removal of reactive oxygen species were more abundant in the extracellular proteome during growth on maltose. In this culture elevated glucose concentrations were observed leading to glucose oxidase (GoxC) synthesis and subsequent oxidation of glucose to gluconic acid and hydrogen peroxide, thus explaining the need for catalases to remove these reactive oxygen species. Late addition of maltose to the culture pregrown on xylose did not lead to elevated levels of extracellular enzymes involved in the removal of reactive oxygen species (e.g. catalases) as the formed glucose resulting from the hydrolysis of maltose will be taken up by the existing fungal biomass and not further oxidized to gluconic acid and hydrogen peroxide, thus making elevated catalase levels dispensable.

### Bioreactor versus shake flask

The major differences influencing fungal physiology in bioreactor versus shake flask culture are related to pH control versus no pH control, good aeration versus bad aeration, and higher mechanical stress due to stirring versus lower mechanical stress due to shaking. This difference in the environmental conditions had a profound effect on the composition of the intracellular proteome. In uncontrolled shake flask cultures, the major glycolytic and TCA cycle enzymes were only present in minor amounts indicating a reduced capacity of the glycolytic pathway and the TCA cycle under these conditions. Moreover, ER-resident chaperones and foldases were present in lower amounts while vacuolar proteases accumulated to higher levels in shake flask culture rendering this culture condition less favourable for recombinant protein production. In this line, previous results also revealed that higher levels of recombinant protein product are generally obtained in bioreactor cultures compared to uncontrolled shake flask cultivations [55,56].

Our results also showed that many proteins found in high abundance under certain culture conditions are still proteins with yet unclear physiological function. Though we are still far from understanding their role, this work now offers more tools for a better understanding of fungal physiology and, thus, for more rational based approaches to improve the productivity of this black fungus.

## Materials and methods

### Strain and medium

*A. niger *AB1.13, an uridine auxotroph, glucoamylase producing strain, was first described as deficient in aspergillopepsin A (PepA) and aspergillopepsin B (PepB) with residual proteolytic activity attributable to the presence of low levels of PepA or the activity of another pepstatin-sensitive protease [42]. *A. niger *AB1.13 was generated by UV mutagenesis of *A. niger *AB4.1 and subsequent selection for reduced extracellular protease activity [42]. *A. niger *AB4.1 is a *pyrG *mutant (requiring uridine or uracil for growth) of *A. niger *N402 [57], which is a *cspA*-derivative (short conidiophores) of *A. niger *ATCC9029 (= NRRL3) [58]. More recently, it was shown that *A. niger *AB1.13 is a regulatory mutant which carries a mutation in the *prtT *gene, a protease specific regulator for the fungal Zn(II)2Cys6 cluster transcription factor family, being responsible for its reduced extracellular proteolytic activity [43].

*A. niger *AB1.13 was grown on a synthetic medium containing either xylose or maltose as carbon source. The defined medium was a modified Vogel' medium [59] supplemented with 2.44 g L^-1 ^uridine. The composition was as follows: Na_3_citrate·2H_2_O, 2.85 g·L^-1^; KH_2_PO_4_, 5 g·L^-1^; NH_4_NO_3_, 2 g·L^-1^; CaCl_2_·2H_2_O, 0.1 g·L^-1^; MgSO_4_·7H_2_O, 0.2 g·L^-1 ^and trace elements (citric acid·H_2_O, 5 mg·L^-1^; ZnSO_4_·7H_2_O, 5 mg·L^-1^; Fe(NH_4_)_2_(SO_4_)_2_·6H2O, 1 mg·L^-1^; CuSO_4_, 0.16 mg·L^-1^; MnSO4·H_2_O, 0.037 mg·L^-1^; H_3_BO_3_, 0.05 mg·L^-1^; Na_2_MoO_4_, 2H_2_O, 0.05 mg·L^-1^). The initial concentration of carbon source, either xylose or maltose, was 20 g·L^-1^. For the study of the change in the composition of the extracellular proteome after induction of *glaA*-promoter by addition of maltose to a culture pregrown on xylose, cultivation was started with an initial concentration 10 g·L^-1 ^xylose and after 68 h of growth on xylose maltose was added to a final concentration of 10 g·L^-1^.

### Inoculation, culture conditions, and analytical procedures

Inoculation was carried out by 1 × 10^7 ^spores mL^-1 ^obtained from a densely conidiating culture grown on 3.8% (w/v) potato dextrose agar (PDA) supplemented with 2.44 g L^-1 ^uridine at 30°C and harvested after 5 days with sterile NaCl solution (0.9% w/v). The spore numbers in the suspension were counted using a hemocytometer.

Shake flask cultivations were carried out in 500 mL Erlenmeyer flasks with 4 baffles on a rotary shaker (Pilot Shaker Rc 6SR, B. Braun Diesel Biotech GmbH, Melsungen, Germany) at 30°C and 120 rpm, and 100 mL working volume. Initial pH was adjusted to 5.5.

Batch cultivations were carried out in a 12 L stirred tank bioreactor Biostat^®^C (B.Braun international) with a working volume of 10 L at 30°C. The stirrer of the Biostat^®^C was equipped with three 6-bladded rushton turbine impellers. The stirrer speed and the aeration rate were 300 rpm and 1 vvm, respectively. The pH was controlled at pH 5.5 using 20% (w/v) NaOH. Foam was suppressed by the manual addition of antifoam reagent Ucolub (Th. Goldschmidt AG, Essen, Germany).

Cell dry mass (CDM) was determined from 10-mL aliquots of culture broth. The fungal biomass was collected by filtration through a filter paper (Sartorius, grade: 389), washed twice with distilled water and dried overnight at 40°C under vacuum to constant weight.

### Preparation of the intracellular protein fraction

Biomass (around 500 mg wet biomass) was harvested by filtration (filter paper: Sartorius, grade: 389) and washed with phosphate buffered saline (PBS) to remove extracellular proteins and other contaminants. Cell disruption was accomplished by grinding the wet biomass in liquid nitrogen using a Mortar Grinder (RM 100, Retsch GmbH &Co. KG, Germany). After cell disruption, the biomass was resuspended in 1 ml extraction buffer (20 mmol L^-1 ^Tris-HCl, pH 7.6, 10 mmol L^-1 ^NaCl, 0.5 mmol L^-1 ^deoxycholate, 1 μg mL^-1 ^pepstatin). Cell debris was removed by centrifugation (22,000 × g, at 4°C for 30 min) and the subsequent supernatant treated for 15 min on ice with 7 μL nuclease mix (0.5 mg mL^-1 ^DNase, 0.25 mg mL^-1 ^RNase, und 50 mmol L^-1 ^MgCl_2_). Then, 10 μL mL^-1 ^sodium deoxycholate (20 mg mL^-1^) were added and the mixture kept for at least 30 min on ice followed by addition of TCA to a final concentration of 12% (w/v). This mixture was incubated for at least 1 h at 4°C and the precipitated proteins were collected by centrifugation at 22,000 × g for 1 h at 4°C. The protein pellet was washed with 300 μL ice cold acetone to remove traces of TCA, centrifuged, washed again with acetone, and solubilized in solubilization buffer containing 7 mol L^-1 ^urea, 2 mol L^-1 ^thiourea, 4% (w/v) CHAPS, 1% (w/v) DTT, 20 mmol L^-1 ^Tris, and 1% (v/v) Pharmalyte™ pH 3-10 (Amersham Biosciences). The total soluble protein concentration was determined according to the method of Bradford [60] using the BIO-RAD protein assay (BIO-RAD Lab., Hartfordshire, USA). The solubilized proteins were stored at -70°C until further analysis by two-dimensional gel electrophoresis.

### Preparation of the extracellular protein fraction

For preparation of the extracellular protein fraction, biomass was removed by centrifugation at 6,000 × g for 15 min at 4°C from the culture broth and the supernatant filtered through a 0.2 μm filter. Protein precipitation, solubilization, and storage were carried out as described above.

### Two-dimensional gel electrophoresis

The first-dimension using isoelectric focusing (IEF) was run with the IPGphor™ Isoelectric Focusing System (Amersham Biosciences) at 20°C with a current of 45 μA per strip. 300 μg of each protein sample were loaded onto Immobiline DryStrip gels of pH 3-10 (IPG strips, Amersham Biosciences) by in-gel rehydration. IEF was performed with the following conditions: 30 V × 12 h, 300 V × 3 h, 600 V × 2 h, 1000 V × 1 h, gradient from 1000 V to 5000 V within 2 h, 5000 V × 2 h, gradient from 5000 V to 8000 V within 2 h, then 8000 V × 10 h. Prior to the second dimension (SDS-PAGE), the IPG strips were equilibrated for 15 min in 6 mol L^-1 ^urea, 30% glycerol (v/v), 2% SDS (w/v), 50 mmol L^-1 ^Tris-HCl pH 8.8, containing 1% (w/v) DTT and then for 15 min in the same buffer additionally containing 2.5% (w/v) iodoacetamide. Equilibrated strips were transferred for the second dimension onto lab cast SDS-polyacrylamide gels (DALT multiple gel caster and DALT gradient maker, Amersham Biosciences). Intra- and extracellular proteins were separated on 12-16% linear gradient and 12% uniform gels, respectively. The second-dimension was carried out using the vertical separation unit Hoefer™ System (Amersham Biosciences) at 10°C in constant working voltage mode as follows: 80 V for 1 h and then 125 V overnight until the bromophenol blue dye front reached the bottom of the gel. Subsequently, gels were stained using colloidal Coomassie Blue G-250 according to the "Blue silver" protocol [61]. The gels were then scanned (ScanMaker 9800 XL, Umax System GmbH, Germany) at 300 dpi resolution to acquire the gel images. Image analysis, namely protein spot detection, matching and quantification were performed using Proteomweaver™ 3.0 (Definiens AG, Germany).

### Identification of protein spots from two-dimensional gels

Protein spots were excised manually from the stained gels. After destaining, reduction and alkylation, in-gel digestion was carried out by incubation with 2 ng μL^-1 ^trypsin (sequencing grade modified, Promega Corp.) in 50 mmol L^-1 ^ammonium bicarbonate at 37°C overnight. Obtained peptides were extracted and then purified with reversed-phased C18 ZipTips (Millipore, USA) as described previously [62]. MALDI-ToF and MALDI ToF/ToF for mass mapping of tryptic peptides were carried out using a Bruker Ultraflex time-of-flight mass spectrometer (Bruker Daltonics GmbH, Germany). Nanoelectrospray ionisation quadrupole-time-of-flight tandem mass spectrometry (ESI-QqTOF MS/MS) was performed using a Q-TOF2 mass spectrometer (Micromass, Manchester, England) to obtain partial amino acid sequences. Peptide mass fingerprints and peptide fragmentation data were processed using FlexAnalysis 2.0 (Bruker Daltonik GmbH, Germany) and the PepSeq program of the software package Masslynx™ (Waters Corporation, USA). The MASCOT 2.1.0 search program (Matrix Science, UK) was used for protein identification with the annotated *Aspergillus niger *genome ([11], EMBL: http://www.ebi.ac.uk/genomes/eukaryota.html) serving as database. The search parameters were set as follows: tryptic digestion, one missed cleavage side allowed, carbamidomethylation of cysteine (fixed modification), methionine oxidation (variable modification), all peptides monoisotopic, peptide tolerance at 20 ppm. All proteins with a Mowse score greater than 54 were regarded as significant (p < 0.05). Annotations are according to the sequenced genome of *A. niger *[11] and the current NCBI Reference Sequence database (http://www.ncbi.nlm.nih.gov/refseq/, status 2010/03/26).

### Nano-HPLC MS/MS

HPLC MS/MS was additionally applied for the analysis of the extracellular proteome of *Aspergillus niger *AB1.13. In this case, proteins were first separated by 12% SDS-PAGE (10 mm distance). After staining with Coomassie brilliant blue, the gel was cut into slices. Protein digestion and peptide isolation was carried out as described for MALDI samples. The separation of the peptide samples was performed using a bioinert Ultimate nano-HPLC system (Dionex, Sunnyvale, CA) as previously described [63]. MS and MS/MS data were acquired using a Q-TOF II mass spectrometer (Waters Corp., Micromass, Manchester, UK). Doubly and triply charged peptide-ions were automatically chosen by the MassLynx software and fragmented for a maximum of 18 s for each peptide. MS data processing and generation of peptide peak lists were also carried out using MassLynx software (Waters Corporation, Manchester, UK). Identification was regarded as valid with a significance value of p < 0.05.

## Competing interests

The authors declare that they have no competing interests.

## Authors' contributions

XL carried out all experimental work and prepared a first draft of the manuscript. JS did the metabolic network analysis and prepared the interactive figures in HTML format, MN and JW contributed to protein identification by Maldi-ToF and Nano-HPLC MS/MS, respectively. APZ was involved in the initial work on network analysis, UR initiated and supervised the study and wrote the final manuscript. All authors read and approved the final manuscript.

## Supplementary Material

Additional file 1 **Biochemical reactions with corresponding enzymes**. Biochemical reactions and corresponding enzymes (coding sequences - CDSs) potentially catalyzing these reactions. Reactions and CDSs depicted in Figure 2 are indicated. Moreover, all CDSs experimentally verified by 2-D GE and MALDI ToF or LC-MS/MS are indicated. Click here for file

Additional file 2 **All identified intracellular proteins**. Classification of intracellular proteins of *A. niger *AB1.13 grown on defined medium with xylose or maltose as carbon substrate that were identified via 2-D GE followed by MALDI ToF. Click here for file

Additional file 3 **Interactive 2-D gel of intracellular proteome of *A. niger *grown on xylose**. Image of 2-D gel for detailed analysis of the intracellular proteome of *A. niger *AB1.13 grown to late exponential/early stationary phase in bioreactor cultures on defined medium with xylose as carbon substrate. The red spots indicate proteins identified by Maldi-ToF analysis. More information on these proteins can be found by moving the mouse over the spots (for locci ID, function, MW and pI, tested with Windows Internet Explorer), or by clicking them to visit the NCBI database http://www.ncbi.nlm.nih.gov/ for their detailed annotation. Please note that the compressed zip file has to be unpacked locally (double click is not sufficient) after downloading. Click here for file

Additional file 4 **Interactive 2-D gel of intracellular proteome of *A. niger *grown on maltose**. Image of 2-D gel for detailed analysis of the intracellular proteome of *A. niger *AB1.13 grown to late exponential/early stationary phase in bioreactor cultures on defined medium with maltose as carbon substrate. The red spots indicate proteins identified by Maldi-ToF analysis. More information on these proteins can be found by moving the mouse over the spots (for locci ID, function, MW and pI, tested with Windows Internet Explorer), or by clicking them to visit the NCBI database http://www.ncbi.nlm.nih.gov/ for their detailed annotation. Please note that the compressed zip file has to be unpacked locally (double click is not sufficient) after downloading. Click here for file

Additional file 5 **Abundance difference of intracellular proteins from xylose and maltose grown cultures**. Identified intracellular proteins showing significant changes in abundance during growth in bioreactor culture using xylose or maltose as carbon substrate. Click here for file

Additional file 6 **All identified extracellular proteins**. Classification of extracellular proteins of *A. niger *AB1.13 grown on defined medium with xylose or maltose as carbon substrate that were identified via 2-D GE followed by MALDI ToF or LC-MS/MS. Click here for file

Additional file 7 **Extracellular proteome of *A. niger *grown on xylose or maltose**. Comparative analysis of the extracellular proteome of *A. niger *AB1.13 grown in bioreactor cultures on defined medium with **(A) **xylose or **(B) **maltose as carbon substrate. Characteristics of proteins indicated by arrows are discussed in more detail in the main body text. The basic side of the gel is on the right. A detailed list of all proteins from the extracellular proteome of *A. niger *growing either on xylose or maltose identified on 2-D gels is found in Additional file 6. Click here for file

Additional file 8 **Abundance difference of intracellular proteins from bioreactor and shake flask grown cultures**. Identified intracellular proteins showing significant changes in abundance during growth on xylose in bioreactor or shake flask culture. Click here for file
