# Supplementary material for: The intra- and extracellular proteome of Aspergillus niger growing on defined medium with xylose or maltose as carbon substrate
Source: Microb Cell Fact. 2010 Apr 20;9:23. doi: 10.1186/1475-2859-9-23 (PMC2874515; doi:10.1186/1475-2859-9-23)
Supplement: Additional file 2 — All identified intracellular proteins. Classification of intracellular proteins of A. niger AB1.13 grown on defined medium with xylose or maltose as carbon substrate that were identified via 2-D GE followed by MALDI ToF. [file 1475-2859-9-23-S2.PDF]

Additional file 2. All identified intracellular proteins. Classification of intracellular proteins of *A. niger* AB1.13 grown on defined medium with xylose or maltose as carbon substrate that were identified via 2-D GE followed by MALDI ToF MS.

Locus ID [1] / Functional classification [1]	NCBI Accession-No [1]	Gene protein [1]	Function and homolog [1]	MW [Da] [2]	pI [3]	Macot Score	Spot-No [4]
**1. Metabolism**							
**1.1 Carbohydrate metabolism**							
**Glycolysis / gluconeogenesis**							
An03g04530	XP_001390334	beta-PGM	Beta-phosphoglucomutase beta-PGM - *L. lactis*	27,128	4.92	70	15
An02g07470	XP_001399881	fba1	Fructose-bisphosphate aldolase Fba1 - *S. cerevisiae*	39,515	5.52	235	39a,b
An14g04920	XP_001401115	**tpiA**	Triose-phosphate-isomerase tpiA from patent WO8704464-A - *A. niger*	26,909	5.6	186	44
An16g01830	XP_001397496	**gpdA**	Glyceraldehyde-3-phosphate dehydrogenase gpdA - *A. niger*	36,187	6.61	194	75
An08g02260	XP_001392342	pgkA	Phosphoglycerate kinase pgkA - *A. nidulans*	44,360	6.17	134	66a,b
An18g06250	XP_001399078	eno1	Phosphopyruvate hydratase ENO1 - *C. albicans*	47,359	5.24	181	27a,b
An07g09530	XP_001392027	pda1	Alpha subunit E1 of the pyruvate dehydrogenase complex Pda1 - *S. cerevisiae*	45,167	7.64	211	81
An07g06840	XP_001391760	lpd1	Precursor of dihydrolipoamide dehydrogenase Lpd1 - *S. cerevisiae*	54,924	8.25	164	86
An17g01530	XP_001398382	**adhA**	Alcohol-dehydrogenase adhA from patent WO8704464-A - *A. niger*	37,132	6.67	244	76
**Citrate cycle (TCA cycle)**							
An08g10530	XP_001393157	aco1	Mitochondrial aconitate hydratase Aco1 - *S. cerevisia*e	84,408	6.18	383	67a,b
An06g00990	XP_001390926	YEL047c	Soluble cytoplasmic fumarate reductase YEL047c - *S. cerevisiae*	51,722	6.08	75	61
An12g07850	XP_001395836	fumR	Fumarate hydratase fumR - *R. oryzae*	57,624	8.11	111	85
An07g02160	XP_001391302	mdh1	Mitochondrial malate dehydrogenase Mdh1 - *S. cerevisiae*	35,735	8.92	262	98a,b
An15g00070	XP_001396546	mdh	Malate dehydrogenase precursor MDH - *M. musculus*	34,378	6.46	262	72
**Pentose phosphate pathway**							
An01g14740	XP_001389862	**goxC, god**	Glucose oxidase precursor goxC - *A. niger*	65,495	4.94	63	16

An02g02930	XP_001399433	rpiB	Ribose-5-phosphate isomerase rpiB - *E. coli*	17,157	7.68	68	82
An07g03850	XP_001391467	tal1	Transaldolase Tal1 - *S. cerevisiae*	35,429	5.57	243	40
**Pyruvate metabolism**							
An16g07110	XP_001398011	ach1	Acetyl-CoA hydrolase Ach1 - *S. cerevisiae*	58,145	6.04	129	60
An14g06340	XP_001401257	rsp29	Hydroxyacylglutathione hydrolase RSP29 - *R. norvegicus*	32,796	6.82	123	78
An08g07290	XP_001392844	**aldA**	Aldehyde dehydrogenase aldA - *A. niger*	53,868	6.15	268	65
An11g01120	XP_001394119	alr	NADPH-dependent aldehyde reductase - *S. salmonicolor*	42,155	6.32	113	71a,b
**Sugar metabolism and others**							
An01g09960	XP_001389416	**xlnD**	Xylosidase xlnD - *A. niger*	87,211	4.76	81	10
An18g03570	XP_001398816	**bgl1**	Beta-glucosidase bgl1 - *A. niger*	93,230	4.64	76	8
An01g03740	XP_001388804	**xyrA**	D-xylose reductase xyrA - *A. niger*	36,095	5.86	97	52
An01g03480	XP_001388778	gutB	Sorbitol dehydrogenase gutB - *B. subtilis*	36,901	5.88	85	53
An01g05800	XP_001389004	estf1	Lactone-specific esterase estf1 - *P. fluorescens*	32,921	8.96	190	100
An01g06970	XP_001389120	ara1	D-arabinose dehydrogenase Ara1 - *S. cerevisiae*	36,746	5.97	188	57
An15g02300	XP_001396769	**abfB**	Arabinofuranosidase B abfB - *A. niger*	52,509	4.23	70	2
An08g01740	XP_001392293	gcy1	Uronate dehydrogenase from patent DE19604798-A1 - *S. cerevisiae*	33,470	6.1	178	64
**1.2 Energy metabolism**							
**Oxidative phosphorylation and ATP synthesis**							
An02g12010	XP_001400329	ipp1	Inorganic pyrophosphatase Ipp1 - *S. cerevisiae*	32,313	5.23	160	24
An01g03570	XP_001388787	mcr1	Cytochrome-b5 reductase Mcr1 - *S. cerevisiae*	36,091	8.93	81	99a,b
An09g06650	XP_001393980	-	Core protein II of ubiquinol-cytochrome c reductase CAA42214.1 - *B. taurus*	47,626	8.83	138	93
An02g04520	XP_001399589	atp5	H+-transporting ATP synthase delta chain precursor Atp5 - *S. cerevisiae*	24,326	9.47	136	102
An15g01710	XP_001396710	atp7	F1Fo-ATP synthase subunit 7 ATP7 - *K. lactis*	19,417	8.73	190	90
**Methane metabolism**							
An18g05480	XP_001398998	aox1	Alcohol oxidase AOX1 - *P. pastoris*	74,594	6.57	159	74a,b
An15g02200	XP_001396759	aod1	Alcohol oxidase AOD1 - *C. boidinii*	65,079	5.44	161	32

An15g00410	XP_001396580	aciA	Acetate-inducible gene aciA - *A. nidulans* [1-3] (aciA = fdh)	39,510	6.08	187	62
**Sulfur metabolism**							
An03g00660	XP_001389970	tauD	Taurine dioxygenase tauD - *E. coli*	41,284	6.1	169	63
An03g02280	XP_001390126	ssuD	FMNH2-dependent aliphatic sulfonate monooxygenase ssuD - *E. coli*	49,929	8.34	65	88
**1.3 Lipid metabolism**							
An02g02960	XP_001399436	acbp	Acyl-CoA-binding type 2 protein Acbp - *S. carlsbergensis*	15,839	5.59	159	43
An04g03360	XP_001401704	aiPLA2	Acidic Ca(2+)-independent phospholipase A2 aiPLA2 - *R. norvegicus*	23,328	5.2	140	22
**1.4 Nucleotide metabolism**							
An07g10100	XP_001392085	adk1	Adenylate kinase Adk1 - *S. cerevisiae*	28,630	8.32	68	87
An02g06030	XP_001399739	uaz	Urate oxidase uaz - *A. flavus*	34,059	6.84	127	79
An02g10320	XP_001400160	nmt1	Protein nmt1 - *A. parasiticus*	38,116	5.81	160	51a,b
An09g05870	XP_001393898	ndk-1	Nucleoside-diphosphate kinase NDK-1 - *N. crassa*	17,008	8.45	86	89
An01g08570	XP_001389279	trxB	Thioredoxin reductase TrxB - *P. chrysogenum*	39,140	5.19	109	21a,b
**1.5 Amino acid metabolism**							
An04g06380	XP_001402002	mAspAT	Mitochondrial aspartate aminotransferase mAspAT - *M. musculus*	47,037	8.92	189	97a,b,c
An02g07430	XP_001399877	YJR139c	Homoserine dehydrogenase YJR139c - *S. cerevisiae*	38,916	5.49	142	36
An02g07500	XP_001399884	lys1	Saccharopine dehydrogenase LYS1 - *C. albicans*	41,382	5.12	118	20
An09g03940	XP_001393710	ilv-2	Ketol-acid reductoisomerase ilv-2 - *N. crassa*	44,559	8.91	166	95
An04g01750	XP_001401543	met6	5-methyltetrahydropteroyltriglutamate-homocysteine S-methyltransferase Met6 - *S. cerevisiae*	87,066	6.23	180	70a,b,c
An08g02700	XP_001392387	eth-1	S-adenosylmethionine synthase eth-1 - *N. crassa*	42,128	5.7	270	47
An02g12060	XP_001400334	cysB	Hypothetical cystathione beta-synthase cysB - *D. discoideum*	19,852	5.57	78	41
**1.6 Metabolism of cofactors and vitamins**							
An11g01630	XP_001394167	nmt2	Thiazole biosynthesis protein nmt2 - *S. pombe*	35,585	5.48	177	35
An14g02460	XP_001400872	**fhbA**	Flavohemoglobin FhbA - *A. niger* [4] (NCBI = fhp = Flavohemoglobin Fhp - *A. eutrophus*)	45,706	5.44	214	33a,b
**1.7 Biodegradation of xenobiotics**							
An03g04560	XP_001390337	morA	Morphine dehydrogenase - *P. putida*	31,387	5.93	55	55

An02g04580	XP_001399596	YAL049c	Hypothetical protein YAL049c - *S. cerevisiae*	27,613	5.99	77	59
**2. Genetic information processing**							
**2.1 Transcription**							
An18g02140	XP_001398673	sbp1p	Spi1-GTPase binding protein sbp1p - *S. pombe*	28,200	4.86	79	12
An01g02880	XP_001388722	cep52	Cytoplasmic ubiquitin / ribosomal fusion protein Cep52 - *S. cerevisiae*	14,554	9.84	78	104
An11g04510	XP_001394443	sup2	Suppressor 2 Sup2 - *S. cerevisiae*	77,978	8.83	62	92
**2.2 Translation**							
An08g03490	XP_001392465	ef-1	Elongation factor 1 beta EF-1 - *O. cuniculus*	33,466	4.55	82	7
**2.3 Sorting and degradation**							
An12g03910	XP_001395465	pinA	Peptidyl-prolyl cis/trans isomerase pinA - *A. nidulans*	19,786	6.18	105	68
An02g14800	XP_001400609	**pdiA**	Protein disulfide isomerase A pdiA - *A. niger*	56,306	4.48	231	6
An04g02020	XP_001401570	cypB	Cyclophilin cypB - *A. nidulans*	29,658	6.55	154	73
An14g00850	XP_001400714	fpr1	Peptidyl-prolyl isomerase Fpr1 - *S. cerevisiae*	11,345	8.79	59	91
An07g08300	XP_001391905	**cypA**	Cyclophilin-like peptidyl prolyl cis-trans isomerase cypA - *A. niger*	18,873	8.87	159	94a,b,c
An12g04940	XP_001395564	hsp60	Mitochondrial heat shock protein Hsp60 - *S. cerevisiae*	61,890	5.51	260	38
An14g04160	XP_001401040	cof1	Cofilin Cof1 - *S. cerevisiae*	17,090	5.47	72	34
An16g09260	XP_001398224	ssb2	DnaK-type molecular chaperone Ssb2 - *S. cerevisiae*	66,884	5.24	185	26
An11g04180	XP_001394413	**bipA**	DnaK-type molecular chaperone bipA - *A. niger*	73,459	4.96	163	18
An05g00810	XP_001390658	TbcA	Tubulin-specific chaperone A TBCA - *O. cuniculus*	13,369	5.57	91	42
An07g05920	XP_001391670	cypD	Estrogen receptor-binding cyclophilin cypD - *B. taurus*	41,129	5.91	188	54
An18g02020	XP_001398661	**tigA**	Disulfide isomerase tigA - *A. niger*	38,592	5.49	128	37a,b
An02g07210	XP_001399855	**pepE**	Aspartic protease pepE - *A. niger*	43,395	4.85	66	11
An08g11190	XP_001393220	ank1	Ankyrin Ank1 - *M. musculus*	97,785	5.67	58	46
An02g01550	XP_001399295	CS-Ag	Secreted serine protease 19 kDa CS antigen CS-Ag - *C. immitis*	15,163	4.24	91	3
An07g03880	XP_001391470	**pepC**	Serine proteinase pepC - *A. niger*	52,803	5.23	72	23

An13g00760	XP_001396216	tpm1	Tropomyosin Tpm1 - *S. cerevisiae*	18,873	4.94	59	17
An12g08760	XP_001395927	vma-4	Vacuolar ATPase subunit E Vma-4 - *N. crassa*	26,037	5.33	188	29
An02g06360	XP_001399773	arc16	Arp2/3 complex 16kD subunit arc16 - *H. sapiens*	20,395	5.43	70	31a,b
**3. Cellular processes (cell cycle and morphogenesis)**							
An14g03180	XP_001400943	cdc3p	Profilin cdc3p - *S. pombe*	13,772	5.72	57	48
An16g05020	XP_001397810	vip1p	Protein vip1p - *S. pombe*	27,778	5.73	118	49
An04g07010	XP_001402062	caM	Calmodulin caM - *A. nidulans*	17,013	4.11	77	1
An01g11960	XP_001389605	bfr1	Brefeldin A resistance protein Bfr1 - *S. cerevisiae*	56,744	8.98	112	101
An02g06710	XP_001399806	mpt4	Suppressor of tom1 protein Mpt4 - *S. cerevisiae*	33,399	9.65	144	103
An14g05320	XP_001401155	wos2p	Cell cycle regulator p21 protein wos2p - *S. pombe*	21,937	4.44	81	5
An02g09550	XP_001400085	het-c2	Protein het-c2 - *P. anserina*	22,105	6.93	68	80
An16g03740	XP_001397686	**pkaR**	cAMP-dependent protein kinase regulatory subunit pkaR - *A. niger*	44,536	4.89	77	14
**4. Others**							
**4.1 Stress response**							
An04g04060	XP_001401773	ccp1	Cytochrome-c peroxidase precursor Ccp1 - *S. cerevisiae*	40,220	7.73	93	83a,b
An16g00920	XP_001397405	pmp20	Peroxisomal membrane protein PMP20 - *C. boidinii*	17,828	5.63	78	45
An01g02500	XP_001388687	-	Thioredoxin - *A. nidulans*	11,914	5.32	82	28
An02g12270	XP_001400355	grrbp2	Glycine-rich RNA-binding protein grrbp2 - *E. esula*	12,842	5.79	68	50a,b
An12g00790	XP_001395168	sti1	Stress-induced protein Sti1 - *S. cerevisiae*	69,875	5.98	119	58
An07g04570	XP_001391539	hex1	Hex1 - *A. nidulans*	24,123	6.76	164	77a,b,c
An01g01830	XP_001388622	cpeB	Catalase/peroxidase cpeB - *S. reticuli*	84,251	6.19	151	69
An07g03770	XP_001391459	sodC	Cu,Zn superoxide dismutase sodC - *A. fumigatus*	15,960	5.94	97	56a,b
An12g08570	XP_001395908	prxII	Type 2 peroxiredoxin PrxII - *B. napus*	18,586	5.38	127	30a,b,c

**4.2 Virulence factors**							
An01g09980	XP_001389418	Asp-HS	Hemolysin Asp-HS - *A. fumigatus*	16,259	7.81	91	84
An01g10050	XP_001389424	IgE-HRF	IgE-dependent histamine-releasing factor - *H. sapiens*	20,275	4.72	65	9
**4.3 Unclassified**							
An02g05620	XP_001399698	-	Hypothetical protein encoded by An07g10060 - *A. niger*	7,972	4.86	82	13
An07g03660	XP_001391449	-	Hypothetical protein CAD37045.1 - *N. crassa* (similar to An09g00630)	28,769	8.91	92	96
An08g10030	XP_001393111	-	Hypothetical MF-7 antigenic protein from patent WO9721817-A1 - *M. sp.*	12,710	5.23	76	25
An09g00630	XP_001393387	cipC	Hypothetical cipC - *A. nidulans*	12,607	5.09	76	19
An09g06410	XP_001393955	-	Hypothetical protein EAA66742.1 - *A. nidulans*	27,897	4.32	98	4a,b

[1] Accession numbers and gene/protein names are according to the sequenced genome of *A. niger* [5] and the NCBI Reference Sequence database (http://www.ncbi.nlm.nih.gov/refseq/). For those proteins annotated as "hypothetical protein" (most proteins of *A.niger*), the similarity information provided in the NCBI annotation in the section "CDS" is shown instead. Genes/proteins in bold are proven genes/proteins of *A. niger*. Functional classification is mostly according to KEGG PATHWAY database (http://www.genome.jp/kegg/metabolism.html). Annotations deviating from the current NCBI Reference Sequence database (status 2010-03-26) are indicated with a reference proving annotation.
[2] Theoretical p*I* not considering potential posttranslational modifications (determined using: http://www.expasy.ch/cgi-bin/pi_tool).
[3] Theoretical average mass not considering potential posttranslational modifications (determined using: http://www.expasy.ch/cgi-bin/pi_tool).
[4] "Spot-No" indicates the serial number of all identified protein spots of the intracellular proteome of *A. niger* AB1.13 from xylose and maltose grown cultures on the interactive and searchable 2-D gels (Additional files 3 and 4, respectively).

References

1 Saleeba JA, Cobbett CS, Hynes MJ: **Characterization of the *amdA*-regulated *aciA* gene of *Aspergillus nidulans*.** *Mol Gen Genet* 1992, **235:** 349-358.

2 Chow CM, RajBhandary UL: **Developmental regulation of the gene for formate dehydrogenase in *Neurospora crassa*.** *J Bacteriol* 1993, **175:** 3703-3709.

3 Tishkov VI, Popov VO: **Catalytic mechanism and application of formate dehydrogenase.** *Biochemistry (Moscow)* 2004, **69:** 1252-1267.

4 te Biesebeke R, Levasseur A, Boussier A, Record E, van den Hondel CA, Punt PJ: **Phylogeny of fungal hemoglobins and expression analysis of the *Aspergillus oryzae* flavohemoglobin gene *fhbA* during hyphal growth.** *Fungal Biol* 2009, **114:** 135-143.

5 Pel HJ, de Winde JH, Archer DB, Dyer PS, Hofmann G, Schaap PJ, Turner G, de Vries RP, Albang R, Albermann K, Andersen MR, Bendtsen JD, Benen JAE, van den Berg M, Breestraat S, Caddick MX, Contreras R, Cornell M, Coutinho PM, Danchin EGJ, Debets AJM, Dekker P, van Dijck PWM, van Dijk A, Dijkhuizen L, Driessen AJM, D'Enfert C, Geysens S, Goosen C, Groot GSP *et al*.: **Genome sequencing and analysis of the versatile cell factory *Aspergillus niger* CBS 513.88.** *Nature Biotechnol* 2007, **25:** 221-231.
