# Supplementary material for: The intra- and extracellular proteome of Aspergillus niger growing on defined medium with xylose or maltose as carbon substrate
Source: Microb Cell Fact. 2010 Apr 20;9:23. doi: 10.1186/1475-2859-9-23 (PMC2874515; doi:10.1186/1475-2859-9-23)
Supplement: Additional file 3 — Interactive 2-D gel of intracellular proteome of A. niger grown on xylose. Image of 2-D gel for detailed analysis of the intracellular proteome of A. niger AB1.13 grown to late exponential/early stationary phase in bioreactor cultures on defined medium with xylose as carbon substrate. The red spots indicate proteins identified by Maldi-ToF analysis. More information on these proteins can be found by moving the mouse over the spots (for locci ID, function, MW and pI, tested with Windows Internet Explorer), or by clicking them to visit the NCBI database http://www.ncbi.nlm.nih.gov/ for their detailed annotation. Please note that the compressed zip file has to be unpacked locally (double click is not sufficient) after downloading. [file 1475-2859-9-23-S3.ZIP › AB113Xyl.html]

Intracellular proteome of Aspergillus niger AB1.13 in xylose medium


**An image of 2-D gel electrophoresis for the intracellular proteome of *Aspergillus niger*  AB1.13 growing in a medium
with *xylose* as carbon source.**
  
  
The red spots indicate those identified by Maldi-ToF analysis. More information can be viewed by moving your mouse over the spots
(for locci ID, function, MW and pI, tested with Windows Internet Explorer), or
by clicking them to visit the NCBI database for their complete annotation.
  
  

**34:cof1**

**40:tal1**

**64:gcy1**

**1:caM**

**2:abfB**

**3:Cs-Ag**

**4a:An09g06410**

**4b:An09g06410**

**5:wos2p**

**6:pdiA;pdi1**

**7:ef-1**

**8:bgl1**

**9:IgE-HRF**

**10:xlnD**

**11:pepE**

**12:sbp1p**

**13:An02g05620**

**14:pkaR**

**15:beta-PGM**

**17:tpm1**

**18:bipA**

**19:cipC**

**20:lys1**

**21a:trxB**

**21b:trxB**

**22:aiPLA2**

**23:pepC**

**24:ipp1**

**25:An08g10030**

**26:ssb2**

**27a:eno1**

**27b:eno1**

**29:vma-4**

**30a:prxII**

**30b:prxII**

**30c:prxII**

**31a:arc16**

**31b:arc16**

**32:aod1**

**33a:fhp**

**33b:fhp**

**35:nmt2p**

**36:YJR139c**

**37a:tigA**

**37b:tigA**

**38:hsp60**

**39a:fba1**

**39b:fba1**

**41:cysB**

**42:tbcA**

**43:acbp**

**44:tpiA**

**45:pmp20**

**47:eth-1**

**48:cdc3p**

**49:vip1p**

**50a:grrbp2**

**50b:grrbp2**

**51a:nmt1**

**51b:nmt1**

**52:xyrA**

**53:gutB**

**54:cypD**

**55:morA**

**56a:sodC**

**56b:sodC**

**57:ara1**

**58:sti1**

**59:YAL049c**

**60:ach1**

**61:YEL047c**

**62:aciA**

**65:aldA**

**66a:pgkA**

**66b:pgkA**

**67a:aco1**

**67b:aco1**

**68:pinA**

**69:cpeB**

**70a:met6**

**70b:met6**

**70c:met6**

**71a:alr**

**71b:alr**

**72:mdh**

**73:cypB**

**74a:aox1**

**75:gpdA**

**76:adhA**

**77a:hex1**

**77b:hex1**

**77c:hex1**

**78:rsp 29**

**79:uaz**

**80:het-c2**

**81:pda1**

**82:rpiB**

**83a:ccp1**

**83b:ccp1**

**84:Asp-HS**

**85:fumR**

**86:lpd1**

**87:adk1**

**88:ssuD**

**89:ndk-1**

**92:sup2**

**93:An09g06650**

**94a:cypA**

**94b:cypA**

**94c:cypA**

**95:ilv-2**

**96:CAD37045.1**

**97a:mAspAT**

**97b:mAspAT**

**97c:mAspAT**

**98a:mdh1**

**98b:mdh1**

**99a:mcr1**

**99b:mcr1**

**100:estf1**

**104:cep52**
