# Supplementary material for: The intra- and extracellular proteome of Aspergillus niger growing on defined medium with xylose or maltose as carbon substrate
Source: Microb Cell Fact. 2010 Apr 20;9:23. doi: 10.1186/1475-2859-9-23 (PMC2874515; doi:10.1186/1475-2859-9-23)
Supplement: Additional file 5 — Abundance difference of intracellular proteins from xylose and maltose grown cultures. Identified intracellular proteins showing significant changes in abundance during growth in bioreactor culture using xylose or maltose as carbon substrate. [file 1475-2859-9-23-S5.PDF]

Additional file 5. Abundance difference of intracellular proteins from xylose and maltose grown cultures. Identified intracellular proteins showing significant changes in abundance during growth in bioreactor culture using xylose or maltose as carbon substrate.

Locus ID<br>Functional classification [1]	Gene/protein [1]	Function and homolog [1]	Fold change [2]<br>(Xylose/maltose)
**Proteins specific to xylose**			
**1. Metabolism**			
**1.1 Carbohydrate metabolism**			
**Sugar metabolism and others**			
An01g09960	**xlnD**	Xylosidase xlnD - *A.niger*	++ (2.571/n.q.) [3]
An01g03740	**xyrA**	D-xylose reductase xyrA - *A. niger*	++ (1.324/n.q.)
An15g02300	**abfB**	Arabinofuranosidase B abfB - *A. niger*	++ (0.735/n.q.)
**Proteins specific to maltose**			
**1. Metabolism**			
**1.1 Carbohydrate metabolism**			
**Pentose phosphate pathway**			
An01g14740	**goxC; god**	Glucose oxidase precursor goxC - *A. niger*	-- (n.q./1.052)
**Proteins showing higher abundance in xylose culture**			
**1. Metabolism**			
**1.1 Carbohydrate metabolism**			
**Pyruvate metabolism**			
An14g06340	rsp29	Hydroxyacylglutathione hydrolase RSP29 - *R. norvegicus*	+2.7 (0.149/0.056)
An11g01120	alr	NADPH-dependent aldehyde reductase - *S. salmonicolor*	+4.2 (3.146/0.756)
**Sugar metabolism and others**			
An18g03570	**bgl1**	Beta-glucosidase bgl1 - *A. niger*	+10.4 (0.763/0.073)
**1.3 Lipid metabolism**			
An04g03360	aiPLA2	Acidic Ca(2+)-independent phospholipase A2 aiPLA2 - *R. norvegicus*	+11.5 (0.620/0.054)
**1.5 Amino acid metabolism**			
An04g01750	met6 [4]	5-methyltetrahydropteroyltriglutamate-homocysteine S-methyltransferase Met6 - *S. cerevisiae*	+2.3 (0.442/0.194)<br>+3.8 (0.319/0.083)
**1.6 Metabolism of cofactors and vitamins**			
An11g01630	nmt2p	Thiazole biosynthesis protein nmt2p - *S. pombe*	+15.2 (1.865/0.123)
**Proteins showing higher abundance in maltose culture**			
**1. Metabolism**			
**1.1 Carbohydrate metabolism**			

**Sugar metabolism and others**			
An01g03480	gutB	Sorbitol dehydrogenase gutB - *B. subtilis*	-2.8 (0.455/1.283)
**2. Genetic information processing**			
**2.3 Sorting and degradation**			
An12g08760	vma-4	Vacuolar ATPase subunit E Vma-4 - *N. crassa*	-2.5 (0.107/0.264)
An02g06360	arc16	Arp2/3 complex 16kD subunit arc16 - *H. sapiens*	-3.3 (0.208/0.684)
**3. Cellular processes (cell cycle and morphogenesis)**			
An16g03740	**pkaR**	cAMP-dependent protein kinase regulatory subunit pkaR - *A. niger*	-2.8 (0.591/1.629)
**4. Others**			
**4.1 Stress response**			
An07g03770	sodC [4]	Cu, Zn superoxide dismutase sodC - *A. fumigatus*	-3.8 (0.124/0.473)<br>-4.2 (0.807/3.415)
An12g08570	prxII [4]	Type 2 peroxiredoxin PrxII - *B. napus*	-3.1 (0.269/0.847)<br>-3.4 (0.420/1.432)<br>-2.8 (1.451/4.112)

[1] Accession numbers and gene/protein names are according to the sequenced genome of *A. niger* [1] and the NCBI Reference Sequence database (http://www.ncbi.nlm.nih.gov/refseq/). For those proteins annotated as "hypothetical protein" (most proteins of *A.niger*), the similarity information provided in the NCBI annotation in the section "CDS" is shown instead. Genes/proteins in bold are proven genes/proteins of *A. niger*. Functional classification is mostly according to KEGG PATHWAY database (http://www.genome.jp/kegg/metabolism.html).

[2] Protein spots were quantified as the normalized spot volume which is the ratio of the single spot volume to the total spots volumes on a 2-D gel. The protein quantities during growth on xylose or maltose are shown in brackets. The left number shows the fold change under respective culture conditions. Only proteins showing more than 2-fold changes are included.

[3] n.q.; Protein spot not quantifiable.

[4] Protein appears in multiple spots.

References

1 Pel HJ, de Winde JH, Archer DB, Dyer PS, Hofmann G, Schaap PJ, Turner G, de Vries RP, Albang R, Albermann K, Andersen MR, Bendtsen JD, Benen JAE, van den Berg M, Breestraat S, Caddick MX, Contreras R, Cornell M, Coutinho PM, Danchin EGJ, Debets AJM, Dekker P, van Dijck PWM, van Dijk A, Dijkhuizen L, Driessen AJM, D'Enfert C, Geysens S, Goosen C, Groot GSP *et al.*: **Genome sequencing and analysis of the versatile cell factory *Aspergillus niger* CBS 513.88.** *Nature Biotechnol* 2007, **25:** 221-231.
