# Supplementary material for: The intra- and extracellular proteome of Aspergillus niger growing on defined medium with xylose or maltose as carbon substrate
Source: Microb Cell Fact. 2010 Apr 20;9:23. doi: 10.1186/1475-2859-9-23 (PMC2874515; doi:10.1186/1475-2859-9-23)
Supplement: Additional file 6 — All identified extracellular proteins. Classification of extracellular proteins of A. niger AB1.13 grown on defined medium with xylose or maltose as carbon substrate that were identified via 2-D GE followed by MALDI ToF or LC-MS/MS. [file 1475-2859-9-23-S6.PDF]

Additional file 6. All identified extracellular proteins. Classification of extracellular proteins of *A. niger* AB1.13 grown on defined medium with xylose or maltose as carbon substrate that were identified via 2-D GE followed by MALDI ToF MS or LC-MS/MS.

Locus ID [1] / Functional classification [1]	NCBI Accession-No [1]	Gene protein [1]	Function and homolog [1]	MW [Da] [2]	pI [3]	Subcellular location	2-D GE	LC-MS/MS
**Extracellular proteins of *A. niger* only found during growth on xylose**								
**1. Metabolism**								
**1.1. Carbohydrate metabolism**								
**5C Sugar hyrolysis**								
An09g03300	XP_001393647	xylS	Alpha-xylosidase XylS - *S. solfataricus*	82,593	5.41	Extracellular/Membrane		+
An01g09960	XP_001389416	**xlnD**	Xylosidase XlnD - *A. niger*	87,211	4.76	Extracellular	+	+
An08g01900	XP_001392309	-	Xylan 1,4-beta-xylosidase - *B. fibrisolvens*	71,092	5.25	Extracellular		+
An01g00780	XP_001388522	**xynB**	Endo-1,4-xylanase XynB - *A. niger*	24,057	5.2	Extracellular	+	+
An03g00940	XP_001389996	**xynA**	Endo-1,4-beta-xylanase A precursor XynA - *A. niger*	35,486	6.17	Extracellular	+	+
An02g10550	XP_001400184	**abnA**	Endo-alpha-1,5-arabinanase abnA - *A. niger*	34,047	4.62	Plasma membrane		+
An03g00960	XP_001389998	**axhA**	1,4-beta-D-arabinoxylan arabinofuranohydrolase AxhA - *A. niger*	35,836	4.62	Extracellular	+	
An15g02300	XP_001396769	**abfB**	Arabinofuranosidase B AbfB - *A. niger*	52,508	4.23	Extracellular	+	
An01g06970	XP_001389120	ara1	D-arabinose dehydrogenase Ara1 - *S. cerevisiae*	36,745	5.97	Cytoplasm	+	
**6C Sugar hydrolysis**								
An01g12150	XP_001389622	**lacA**	Beta-galactosidase lacA - *A. niger*	109,712	5.05	Extracellular		+
An02g11150	XP_001400244	**aglB**	Alpha-galactosidase AglB - *A. niger*	48,826	4.89	Extracellular	+	+
An16g06800	XP_001397982	**eglB**	Endoglucanase EglB - *A. niger*	45,254	4.42	Extracellular		+
An14g02760	XP_001400902	**eglA**	Endoglucanase A EglA - *A. niger*	25,873	4.49	Extracellular	+	+
An08g03580	XP_001392475	bgt1	1,3-beta-glucanosyltransferase Bgt1 - *A. fumigatus*	32,091	4.54	Cell wall	+	+
An03g05290	XP_001390410	bgl2	Glucan 1,3-beta-glucosidase Bgl2 - *S. cerevisiae*	46,806	4.39	Extracellular/Membrane		+
An14g05800	XP_001401203	**aguA**	Alpha-glucuronidase AguA - *A. niger*	93,732	5.17	Extracellular		+

An01g12550	XP_001389661	msdS	Mannosyl-oligosaccharide 1,2-alpha-mannosidase MsdS - *A. saitoi*	55,928	4.83	Extracellular		+
**Other carbohydrate metabolism**								
An02g09090	XP_001400045	-	Mutarotase enzyme - *S. scrofa*	44,139	4.59	Extracellular		+
An18g04100	XP_001398868	gp43	Secreted glycoprotein precursor Gp43 - *P. brasiliensis*	45,525	4.97	Extracellular		+
An02g05730	XP_001399709	bcsB	Cellulose synthase protein BcsB - *A. xylinum*	57,517	7.17	Extracellular		+
An10g00400	XP_001402433	gel1	1,3-beta-glucanosyltransferase Gel1 - *A. fumigatus*	48,465	4.73	Extracellular		+
An09g00120	XP_001393337	**faeA**	Ferulic acid esterase A FaeA - *A. niger*	30,549	4.37	Extracellular/Membrane	+	+
**1.2. Amino acid metabolism**								
An04g06380	XP_001402002	mAspAT	Mitochondrial aspartate aminotransferase mAspAT - *M. musculus*	47,036	8.92	Mitochondria	+	
An04g00990	XP_001401464	**gdhA**	NADP-dependent glutamate dehydrogenase GdhA - *A. niger*	49,376	5.79	Mitochondria	+	
An03g03660	XP_001390247	glr1	Glutathione reductase Glr1 - *S. cerevisiae*	51,652	5.93	Cytoplasm/Membrane	+	
**1.3. Metabolism of cofactors and vitamins**								
An03g00460	XP_001389952	-	6-hydroxy-D-nicotine oxidase 6-HDNO - *A. oxidans*	54,709	5.05	Extracellular		+
An18g02690	XP_001398728	DHGO	Dihydrogeodin oxidase DHGO - *A. terreus*	72,473	5.64	?????		+
**2. Others**								
**Cell wall proteins**								
An01g11010	XP_001389518	crh1	Cell wall protein Crh1 - *S. cerevisiae*	39,886	4.06	Cell wall		+
An14g01820	XP_001400808	binB	Hypothetical cell wall protein BinB - *A. nidulans*	19,180	4.87	Extracellular/Membrane		+
**Signal transduction**								
An01g14940	XP_001389882	-	Nonhemolytic phospholipase C - *B. pseudomallei*	49,683	5.11	Extracellular		+
**Unclassified proteins**								
An01g12240	XP_001389631	-	Hypothetical protein CAC28784.2 - *N. crassa*	27,089	4.97	Extracellular		+
An04g08150	XP_001402173	-	EST an_2637 - *A. niger*	17,131	6.06	Nucleus		+
An02g11390	XP_001400266	-	Hypothetical protein - *D. radiodurans*	86,208	4.62	Extracellular		+
An11g01120	XP_001394119	alr	NADPH-dependent aldehyde reductase - *S. salmonicolor*	42,155	6.32	?????	+	+

Extracellular proteins of *A. niger* only found during growth on maltose

1. Metabolism

1.1. Carbohydrate metabolism

6C sugar hydrolysis

An11g03340	XP_001394335	**amyA**	Acid alpha-amylase - *A. niger*	55,238	4.25	Extracellular/Membrane	+	+
An02g04900	XP_001399628	**pgaB**	Endopolygalacturonases PgaB - *A. niger*	37,819.	6.31	Extracellular		+
An01g12450	XP_001389652	exgS	Glucan beta-1,3 exoglucanase ExgS - *A. phoenicis*	98,797	4.53	Extracellular/Membrane		+
An04g06920	XP_001402053	**aglU**	Extracellular alpha-glucosidase aglU - *A. niger*	108,914	5.14	Extracellular		+
An02g13580	XP_001400489	-	Endochitinase from patent EP531218-A - *A. album*	43,084	6.59	?????		+

Glycolysis, pentose phosphate pathway and TCA cycle

An12g08610	XP_001395912	**glkA**	Glucokinase GlkA - *A. niger*	54,507	5.45	Cytoplasm		+
An16g05420	XP_001397850	pgi1	Glucose-6-phosphate isomerase Pgi1 - *S. cerevisiae*	61,555	5.98	Cytoplasm/Membrane		+
An16g01830	XP_001397496	**gpdA**	Glyceraldehyde-3-phosphate dehydrogenase GpdA - *A. niger*	36,187	6.61	Cytoplasm		+
An09g06680	XP_001393983	**citA**	Citrate synthase citA - *A. niger*	52,025	8.57	Mitochondria		+
An01g14740	XP_001389862	**goxC**	Glucose oxidase precursor GoxC - *A. niger*	65,495	4.94	Extracellular		+

1.2. Energy metabolism

An02g01830	XP_001399324	**cyc**	cytochrome C Cyc - *A. niger*	12,032	9.28	Mitochondria		+

1.3. Amino acid metabolism

An16g05570	XP_001397865	-	Aspartate transaminase - *S. scrofa*	46,492	8.53	?????		+

1.4. Phosphate metabolism

An12g01910	XP_001395277	phyA3	Phytase PhyA3 - *A. fumigatus*	53,875	4.74	Extracellular		+

2. Genetic information processing

2.1 Sorting and degradation

An01g02880	XP_001388722	cep52	Cytoplasmic ubiquitin / ribosomal fusion protein - *S. cerevisiae*	14,554	9.84	Cytoplasm		+
An14g04710	XP_001401093	**pepA**	Aspartic proteinase aspergillopepsin I PepA - *A. niger*	41,276	4.46	Extracellular	+	
An03g01660	XP_001390068	ape3	Vacuolar aminopeptidase Y ape3 - *S. cerevisiae*	59,349	4.74	Vacuole	+	
An03g05200	XP_001390401	-	Carboxypeptidase S1 - *P. janthinellum*	62,599	4.57	Extracellular		+
An07g08300	XP_001391905	**cypA**	Cyclophilin-like peptidyl prolyl cis-trans isomerase CypA - *A. niger*	18,873	8.87	Cytoplasm		+

3. Others

Signal transduction

An16g04200	XP_001397731	rho2P	GTP-binding protein Rho2p - *S. pombe*	21,904	5.8	Plasma membrane	+	

**Stress response/detoxification**								
An01g01820	XP_001388621	**catR**	Catalase R catR - *A. niger*	80,495	5.5	Peroxisome	+	
An12g09510	XP_001396003	merA	Mercury(II) reductase MerA - *Bacillus sp.*	49,562	6.24	?????		+
An12g08570	XP_001395908	prixII	Type 2 peroxiredoxin PrxII - *B. napus*	18,586	5.38	Peroxisome	+	+
An16g00920	XP_001397405	pmp20	Peroxisomal membrane protein PMP20 - *C. boidinii*	17,828	5.63	Peroxisome	+	

Extracellular proteins of *A. niger* found during growth on xylose and maltose

**1. Metabolism**								
**1.1 Carbohydrate metabolism**								
**6C sugar hydrolysis**								
An03g06550	XP_001390530	**glaA**	Glucan 1,4-alpha-glucosidase, Glucoamylase glaA - *A. niger*	68,308	4.25	Extracellular	+	+
An18g03570	XP_001398816	**bgl1**	Beta-glucosidase bgl1 - *A. niger*	93,229	4.64	Extracellular	+	+
An02g07020	XP_001399836	cts1	Chitinase 1 precursor cts1 - *C. immitis*	45,349	4.91	Extracellular	+	
**1.2 Amino acid metabolism**								
An01g09220	XP_001389342	melC2	Tyrosinase melC2 - *S. lincolnensis*	44,675	5.55	Extracellular		+
An14g02470	XP_001400873	-	Protein PRO304 - *Homo sapiens*	65,699	5.38	Extracellular		+
An01g14730	XP_001389861	TcMLE	Cis,cis-muconate lactonizing enzyme I TcMLE - *T. cutaneum*	41,645	5.45	Extracellular	+	+
**2. Others**								
**Sorting and transport**								
An14g05020	XP_001401125	vac8	Armadillo repeat-containing protein vac8 - *S. cerevisiae*	62,544	4.91	Vacuole		+
**Stress response/detoxification**								
An01g01550	XP_001388595	cat1	Catalase cat1 - *A. fumigatus*	80,216	5.11	?????	+	+
**Virulence and disease factors**								
An07g03340	XP_001391417	hYp1	Hydrophobin hYP1 - *A. fumigatus*	16,225	4.23	Extracellular/Membrane		+
An01g09980	XP_001389418	Asp-HS	Hemolysin Asp-HS - *A. fumigatus*	16259	7.81	Extracellular	+	
**Unclassified proteins**								
An09g05940	XP_001393905	**sox**	Sulphydryl oxidase Sox - *A. niger*	43,498	5.44	Extracellular/Membrane	+	+
An02g07520	XP_001399886	trpE	Hypothetical anthranilate synthase trpE - *B. halodurans*	47,457	9.13	Mitochondria		+

An18g02752	XP_001398735	-	Hypothetical protein BAC47708.1 - *B. japonicum*	41,935	4.89	Extracellular	+

[1] Accession numbers and gene/protein names are according to the sequenced genome of *A. niger* [1] and the NCBI Reference Sequence database (http://www.ncbi.nlm.nih.gov/refseq/). For those proteins annotated as "hypothetical protein" (most proteins of *A.niger*), the similarity information provided in the NCBI annotation in the section "CDS" is shown instead. Genes/proteins in bold are proven genes/proteins of *A. niger*. Functional classification is mostly according to KEGG PATHWAY database (http://www.genome.jp/kegg/metabolism.html).
[2] Theoretical p*I* not considering potential posttranslational modifications (determined using: http://www.expasy.ch/cgi-bin/pi_tool).
[3] Theoretical average mass of the polypeptide chain not considering potential posttranslational modifications (determined using: http://www.expasy.ch/cgi-bin/pi_tool).

References

1 Pel HJ, de Winde JH, Archer DB, Dyer PS, Hofmann G, Schaap PJ, Turner G, de Vries RP, Albang R, Albermann K, Andersen MR, Bendtsen JD, Benen JAE, van den Berg M, Breestraat S, Caddick MX, Contreras R, Cornell M, Coutinho PM, Danchin EGJ, Debets AJM, Dekker P, van Dijck PWM, van Dijk A, Dijkhuizen L, Driessen AJM, D'Enfert C, Geysens S, Goosen C, Groot GSP *et al*.: **Genome sequencing and analysis of the versatile cell factory *Aspergillus niger* CBS 513.88.** *Nature Biotechnol* 2007, **25:** 221-231.
