# Supplementary material for: The intra- and extracellular proteome of Aspergillus niger growing on defined medium with xylose or maltose as carbon substrate
Source: Microb Cell Fact. 2010 Apr 20;9:23. doi: 10.1186/1475-2859-9-23 (PMC2874515; doi:10.1186/1475-2859-9-23)
Supplement: Additional file 7 — Extracellular proteome of A. niger grown on xylose or maltose. Comparative analysis of the extracellular proteome of A. niger AB1.13 grown in bioreactor cultures on defined medium with (A) xylose or (B) maltose as carbon substrate. Characteristics of proteins indicated by arrows are discussed in more detail in the main body text. The basic side of the gel is on the right. A detailed list of all proteins from the extracellular proteome of A. niger growing either on xylose or maltose identified on 2-D gels is found in Additional file 6. [file 1475-2859-9-23-S7.PDF]

Additional file 7

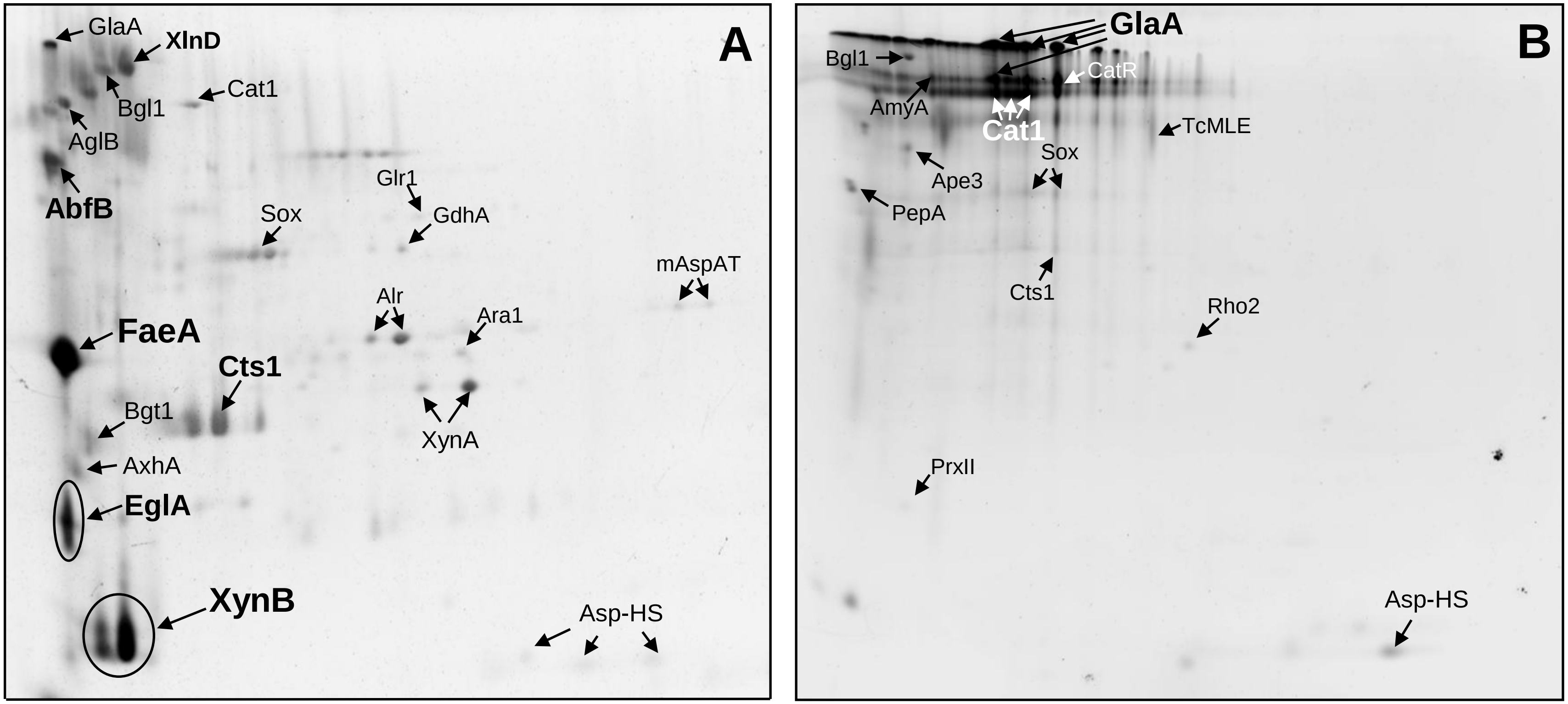

Extracellular proteome of *A. niger* grown on xylose or maltose. Comparative analysis of the extracellular proteome of *A. niger* AB1.13 grown in bioreactor cultures on defined medium with **(A)** xylose or **(B)** maltose as carbon substrate. Characteristics of proteins indicated by arrows are discussed in more detail in the main body text. The basic side of the gel is on the right. A detailed list of all proteins from the extracellular proteome of *A. niger* growing either on xylose or maltose identified on 2-D gels is found in Additional file 6.
