# Supplementary material for: The intra- and extracellular proteome of Aspergillus niger growing on defined medium with xylose or maltose as carbon substrate
Source: Microb Cell Fact. 2010 Apr 20;9:23. doi: 10.1186/1475-2859-9-23 (PMC2874515; doi:10.1186/1475-2859-9-23)
Supplement: Additional file 8 — Abundance difference of intracellular proteins from bioreactor and shake flask grown cultures. Identified intracellular proteins showing significant changes in abundance during growth on xylose in bioreactor or shake flask culture. [file 1475-2859-9-23-S8.PDF]

Additional file 8. Abundance difference of intracellular proteins from bioreactor and shake flask grown cultures. Identified intracellular proteins showing significant changes in abundance during growth on xylose in bioreactor or shake flask culture.

**Locus ID**<br>**Functional classification** [1]	**Gene/ protein** [1]	**Function and homolog** [1]	**Fold change** [2]<br>**(Bioreactor/shake flask)**
**1. Metabolism**			
**1.1 Carbohydrate metabolism**			
**Glycolysis / gluconeogenesis**			
An03g04530	beta-PGM	Beta-phosphoglucomutase beta-PGM - *L. lactis*	++ (0.398/n.q.) [3]
An02g07470	fba1 [4]	Fructose-bisphosphate aldolase Fba1 - *S. cerevisiae*	++ (2.957/n.q.)<br>+1.5 (1.466/0.993)
An14g04920	**tpiA**	Triose-phosphate-isomerase tpiA - *A. niger*	+3.3 (2.003/0.614)
An16g01830	**gpdA**	Glyceraldehyde-3-phosphate dehydrogenase gpdA - *A. niger*	+4.3 (4.054/0.952)
An18g06250	eno1 [4]	Phosphopyruvate hydratase ENO1 - *C. albicans*	+3.3 (0.383/0.116)<br>+3.6 (0.609/0.169)
An07g09530	pda1	Alpha subunit E1 of the pyruvate dehydrogenase complex Pda1 - *S. cerevisiae*	+2.4 (0.543/0.226)
An07g06840	lpd1	Dihydrolipoamide dehydrogenase Lpd1 - *S. cerevisiae*	+4.5 (0.481/0.106)
**Citrate cycle (TCA cycle)**			
An08g10530	**aco1** [4]	Mitochondrial aconitate hydratase Aco1 - *S. cerevisiae*	+1.6 (0.139/0.086)<br>++ (0.438/n.q.)
An06g00990	YEL047c	Cytoplasmic fumarate reductase YEL047c - *S. cerevisiae*	+2.5 (0.280/0.110)
An12g07850	fumR	Fumarate hydratase fumR - *R. oryzae*	++ (0.375/n.q.)
An07g02160	mdh1 [4]	Mitochondrial malate dehydrogenase Mdh1 - *S. cerevisiae*	+4.4 (4.712/1.064<br>+27.5 (5.502/0.200)
An15g00070	mdh	Malate dehydrogenase precursor MDH - *M. musculus*	+4.8 (1.998/0.419)
**Pentose phosphate pathway**			
An07g03850	tal1	Transaldolase Tal1 - *S. cerevisiae*	+5.2 (1.309/0.251)
**Pyruvate metabolism**			
An16g07110	ach1	Acetyl-CoA hydrolase Ach1 - *S. cerevisiae*	+5.7 (0.838/0.147)
An11g01120	alr	NADPH-dependent aldehyde reductase - *S. salmonicolor*	+2.1 (3.858/1.827)
**Sugar metabolism and others**			
An01g09960	**xlnD**	xylosidase xlnD - *A. nige*r	+2.1 (3.158/1.519)
An01g03740	**xyrA** [4]	D-xylose reductase xyrA - *A. niger*	+2.1 (1.626/0.784)<br>+2.1 (1.844/0.872)
**1.2 Energy Metabolism**			
**Oxidative phosphorylation and ATP synthesis**			
An02g12010	ipp1	Inorganic pyrophosphatase Ipp1 - *S. cerevisiae*	+5.7 (0.946/0.167)
**Methane metabolism**			
An15g00410	aciA	Acetate-inducible gene aciA - *A. nidulans*	-2.0 (0.722/1.420)

**Sulfur metabolism**			
An03g00660	tauD	Taurine dioxygenase tauD - *E. coli*	+2.4 (0.539/0.229)
**1.3 Lipid metabolism**			
An04g03360	aiPLA2	Acidic Ca(2+)-independent phospholipase A2 - *R. norvegicus*	++ (0.739/n.q.)
**1.4 Nucleotide metabolism**			
An07g10100	adk1 [4]	Adenylate kinase Adk1 - *S. cerevisiae*	-2.5 (0.352/0.881)<br>-6.0 (0.185/1.108)
An02g10320	nmt1	Protein nmt1 - *A. parasiticus*	++ (1.220/n.q.)
An01g08570	trxB [4]	Thioredoxin reductase TrxB - *P. chrysogenum*	+ 2.4 (0.579/0.243)<br>+1.1 (0.286/0.254)
**1.5 Amino acid metabolism**			
An02g07500	lys1	Saccharopine dehydrogenase LYS1 - *C. albicans*	++ (0.526/n.q.)
An04g01750	met6 [4]	5-methyltetrahydropteroyltriglutamate-homocysteine S-methyltransferase Met6 - *S. cerevisiae*	+2.3 (0.543/0.236)<br>+2.4 (0.392/0.162)
**1.6 Metabolism of cofactors and vitamins**			
An11g01630	nmt2p	Thiazole biosynthesis protein nmt2p - *S. pombe*	++ (2.291/n.q.)
An14g02460	**fhbA** [4]	Flavohemoglobin FhbA - *A. niger* [1]<br>(NCBI = fhp = Flavohemoglobin Fhp - *A. eutrophus*)	+6.9 (2.900/0.420)<br>++ (1.205/n.q)
**2. Genetic information processing**			
**2.3 Sorting and degradation**			
An02g14800	**pdiA**	Protein disulfide isomerase A pdiA - *A. niger*	+2.4 (1.512/0.627)
An07g08300	**cypA** [4]	Cyclophilin-like peptidyl prolyl cis-trans isomerase - *A. niger*	+5.1 (1.988/0.389)<br>+1.4 (2.265/1.612)
An12g04940	hsp60 [4]	Mitochondrial heat shock protein Hsp60 - *S. cerevisiae*	+3.0 (0.698/0.230)<br>+4.0 (0.726/0.181)
An16g09260	ssb2	DnaK-type molecular chaperone Ssb2 - *S. cerevisiae*	+5.2 (1.413/0.270)
An11g04180	**bipA**	DnaK-type molecular chaperone bipA - *A. niger*	++ (0.451/n.q.)
An05g00810	TbcA	Tubulin-specific chaperone A TBCA - *O. cuniculus*	-3.1 (0.177/0.557)
An18g02020	**tigA** [4]	Disulfide isomerase tigA - *A. niger*	+2.8 (1.111/0.392)<br>+1.2 (0.799/0.659)
An02g07210	**pepE**	Aspartic protease pepE - *A. niger*	-10.5 (0.616 /6.438)
**3. Cellular processes (cell cycle and morphogenesis)**			
An04g07010	caM	Calmodulin caM - *A. nidulans*	-2.6 (1.057/2.756)
An01g11960	bfr1	Brefeldin A resistance protein Bfr1 - *S. cerevisiae*	-- (n.q./0.180)
An02g06710	mpt4	Suppressor of tom1 protein Mpt4 - *S. cerevisiae*	-- (n.q. /0.287)
An14g05320	wos2p	Cell cycle regulator p21 protein wos2p - *S. pombe*	-2.4 (0.450/1.096)
**4. Others**			
**4.1 Stress response**			
An07g04570	hex1 [4]	hex1 - *A. nidulans*	-4.6 (0.080/0.370)<br>-2.7 (0.459/1.229)
An07g03770	sodC [4]	Cu,Zn superoxide dismutase sodC - *A. fumigatus*	-3.3 (0.992/3.259)

			-- (n.q./1.324)
**4.2 Virulence factors**			
An01g09980	Asp-HS	hemolysin Asp-HS - *A. fumigatus*	-3.0 (0.811/2.412)
**4.3 Unclassified**			
An02g05620	-	Hypothetical protein encoded by An07g10060 - *A. niger*	-4.3 (0.499/2.156)
An07g03660	-	Hypothetical protein CAD37045.1 - *N. crassa* (similar to An09g00630)	-8.5 (0.591/4.998)
An09g06410	-	Hypothetical protein EAA66742.1 - *A. nidulans*	-4.6 (0.363/1.673)

[1] Accession numbers and gene/protein names are according to the sequenced genome of *A. niger* [2] and the NCBI Reference Sequence database (http://www.ncbi.nlm.nih.gov/refseq/). For those proteins annotated as "hypothetical protein" (most proteins of *A.niger*), the similarity information provided in the NCBI annotation in the section "CDS" is shown instead. Genes/proteins in bold are proven genes/proteins of *A. niger*. Functional classification is mostly according to KEGG PATHWAY database (http://www.genome.jp/kegg/metabolism.html). Annotations deviating from the current NCBI Reference Sequence database (status 2010-03-26) are indicated with a reference proving annotation
[2] Protein spots were quantified as the normalized spot volume which is the ratio of the single spot volume to the total spots volumes on a 2-D gel. The protein quantities during growth on xylose in bioreactor or shake flask culture are shown in brackets. The left number shows the fold change of this protein in bioreactor culture relative to shake flask culture. Only proteins showing more than 2-fold changes are included.
[3] n.q.; Protein spot not quantifiable.
[4] Protein appears in multiple spots.

References

1 te Biesebeke R, Levasseur A, Boussier A, Record E, van den Hondel CAMJJ, Punt PJ: **Phylogeny of fungal hemoglobins and expression analysis of the *Aspergillus oryzae* flavohemoglobin gene *fhbA* during hyphal growth.** *Fungal Biol* 2010, **114:** 135-143.

2 Pel HJ, de Winde JH, Archer DB, Dyer PS, Hofmann G, Schaap PJ, Turner G, de Vries RP, Albang R, Albermann K, Andersen MR, Bendtsen JD, Benen JAE, van den Berg M, Breestraat S, Caddick MX, Contreras R, Cornell M, Coutinho PM, Danchin EGJ, Debets AJM, Dekker P, van Dijck PWM, van Dijk A, Dijkhuizen L, Driessen AJM, D'Enfert C, Geysens S, Goosen C, Groot GSP *et al.*: **Genome sequencing and analysis of the versatile cell factory *Aspergillus niger* CBS 513.88.** *Nature Biotechnol* 2007, **25:** 221-231.
